# 2D Ferromagnetic M_3_GeTe_2_ (M = Ni/Fe) for Boosting Intermediates Adsorption toward Faster Water Oxidation

**DOI:** 10.1002/advs.202310115

**Published:** 2024-03-16

**Authors:** Guyue Bo, Peng Li, Yameng Fan, Xiaobo Zheng, Mengting Zhao, Qiang Zhu, Yang Fu, Yitong Li, Wei Kong Pang, Wei Hong Lai, Bernt Johannessen, Lars Thomsen, Bruce Cowie, Tianyi Ma, Cheng Wang, Guan Heng Yeoh, Yi Du, Shi Xue Dou, Xun Xu

**Affiliations:** ^1^ Institute for Superconducting & Electronic Materials Australian Institute for Innovative Materials University of Wollongong Wollongong NSW 2522 Australia; ^2^ School of Science RMIT University Melbourne VIC 3000 Australia; ^3^ School of Physics and Astronomy Monash University Clayton VIC 3800 Australia; ^4^ Electron Microscopy Center University of Wollongong Wollongong NSW 2500 Australia; ^5^ Australian Synchrotron Australian Nuclear Science and Technology Organization Clayton VIC 3168 Australia; ^6^ School of Mechanical and Manufacturing Engineering University of New South Wales Sydney NSW 2052 Australia; ^7^ School of Physics and BUAA‐UOW Joint Research Centre Beihang University Beijing 100191 P. R. China; ^8^ Institute of Energy Materials Science (IEMS) University of Shanghai for Science and Technology 516 Jungong Road Shanghai 200093 P. R. China

**Keywords:** 2D ferromagnetic, atomic defects, hydroxyl adsorption, Ni_3_/Fe_3_GeTe_2_, oxygen evolution reaction

## Abstract

In this work, 2D ferromagnetic M_3_GeTe_2_ (MGT, M = Ni/Fe) nanosheets with rich atomic Te vacancies (2D‐MGT_v_) are demonstrated as efficient OER electrocatalyst via a general mechanical exfoliation strategy. X‐ray absorption spectra (XAS) and scanning transmission electron microscope (STEM) results validate the dominant presence of metal‐O moieties and rich Te vacancies, respectively. The formed Te vacancies are active for the adsorption of OH* and O* species while the metal‐O moieties promote the O* and OOH* adsorption, contributing synergistically to the faster oxygen evolution kinetics. Consequently, 2D‐Ni_3_GeTe_2v_ exhibits superior OER activity with only 370 mV overpotential to reach the current density of 100 mA cm^−2^ and turnover frequency (TOF) value of 101.6 s^−1^ at the overpotential of 200 mV in alkaline media. Furthermore, a 2D‐Ni_3_GeTe_2v_‐based anion‐exchange membrane (AEM) water electrolysis cell (1 cm^2^) delivers a current density of 1.02 and 1.32 A cm^−2^ at the voltage of 3 V feeding with 0.1 and 1 m KOH solution, respectively. The demonstrated metal‐O coordination with abundant atomic vacancies for ferromagnetic M_3_GeTe_2_ and the easily extended preparation strategy would enlighten the rational design and fabrication of other ferromagnetic materials for wider electrocatalytic applications.

## Graphical abstract

2D ferromagnetic M_3_GeTe_2_ (MGT, M = Ni/Fe) nanosheets with rich atomic Te vacancies are experimentally prepared as efficient electrocatalysts for the alkaline OER, where the generated atomic vacancies and surface metal‐oxygen configurations are crucial for the highly improved intermediates adsorption, charge transfer kinetics, and overall reaction performances, enlighten the rational design of broad ferromagnetic materials for a wide range of electrocatalytic applications.

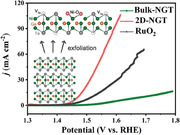

## 1 Introduction

Anion‐exchange membrane (AEM) water electrolyzer is a very promising technology for producing green hydrogen (H_2_) with the input of renewable electricity, water, and abundant catalysts.^[^ ^1^, ^2^, ^3^, ^4^, ^5^, ^6^, ^7^ ^]^ Compared to the conventional proton‐exchange membrane (PEM) water electrolyzers and alkaline water electrolyzers, AEM water electrolyzers feature many appealing advantages including significantly reduced production costs of major cell components (bipolar plates and membrane), fewer corrosion problems, and easy deployment.^[^ ^3^, ^8^, ^9^, ^10^, ^11^, ^12^, ^13^ ^]^ Of the two fundamental reactions for the AEM water electrolyzer, the oxygen evolution reaction (OER) is a four‐electron‐transfer electrode reaction accompanied by the adsorption of multiple intermediates and O─O bond formation.^[^ ^14^, ^15^ ^]^ The high energy barrier associated with intermediates adsorption (e.g., OH*, O*, OOH*) and O─O bond formation often leads to significant overpotential to drive the reaction to happen. Consequently, previous catalyst design strategies usually focus on using noble metals and creating various noble metal nanoparticles, metal alloys, heterostructures, etc, as efficient catalysts. Noble metal‐based electrocatalysts have decent activity; however, the ultimate goal of AEM water electrolyzers is to achieve the deployment of non‐noble metal and/or metal‐free compounds as electrocatalysts to improve the capital cost of the electrolyzers and hydrogen production cost. Meanwhile, the scarcity and high production cost of noble metals limit their long‐term applications. Therefore, developing efficient electrocatalysts from earth‐abundant elements is critical to increase the competitiveness of hydrogen production costs.

Recently, 2D ferromagnetic materials M_3_GeTe_2_ (M = Ni/Fe, denoted as MGT,) as a typical example of a topological quantum material, have attracted wider attention in the fields of topology, spintronics, and electrocatalysis owing to their giant electron motilities, metallic character, topological electronic band structures, and high stability.^[^ ^16^, ^17^, ^18^ ^]^ The relatively weak van der Waals forces for MGT make the preparation of thin‐layered MGT possible upon layer exfoliation, where the physiochemical character could be rather interesting at an atomic level.^[^ ^17^, ^19^, ^20^ ^]^ Meanwhile, by using theoretical simulations, several studies speculated the catalytic activity of M_3_GeTe_2_ in nitrogen reduction reaction, sulfur reduction reaction, and even oxygen evolution reaction.^[^ ^17^, ^21^, ^22^, ^23^ ^]^ However, a critical demonstration from the viewpoint of experimental research has rarely been reported. More importantly, lots of atomic vacancies, surface reconstruction, and phase evolution are very likely to occur, which are distinct from theoretical studies and deserve to be re‐investigated given their unique role in activating intermediate adsorption in these electrocatalytic reactions.^[^ ^24^, ^25^, ^26^ ^]^

To this end, as a proof‐of‐concept, M_3_GeTe_2_ (M = Ni/Fe, denoted as MGT) was fabricated as a bulk material by a solid‐state reaction. Subsequently, 2D MGT nanosheets with rich atomic vacancies (2D‐MGT_v_) were successfully acquired by using a general one‐step liquid exfoliation method. With the use of a series of experimental techniques (SEM, TEM, XPS, and XAS), it is interesting to find out that the 2D‐MGT_v_ features a very high proportion of Te vacancies and metal‐O moieties but still maintains a metallic character compared to its bulk counterparts. We further employed the oxygen evolution reaction as an example to examine how the change of MGT structures is supposed to affect the catalytic activity. Our results suggest that 2D‐MGT_v_ is a very promising OER electrocatalyst, and its fascinating structure endows it with rather broad electrochemical applications.

## 2 Results and Discussion

**Figure 1:**
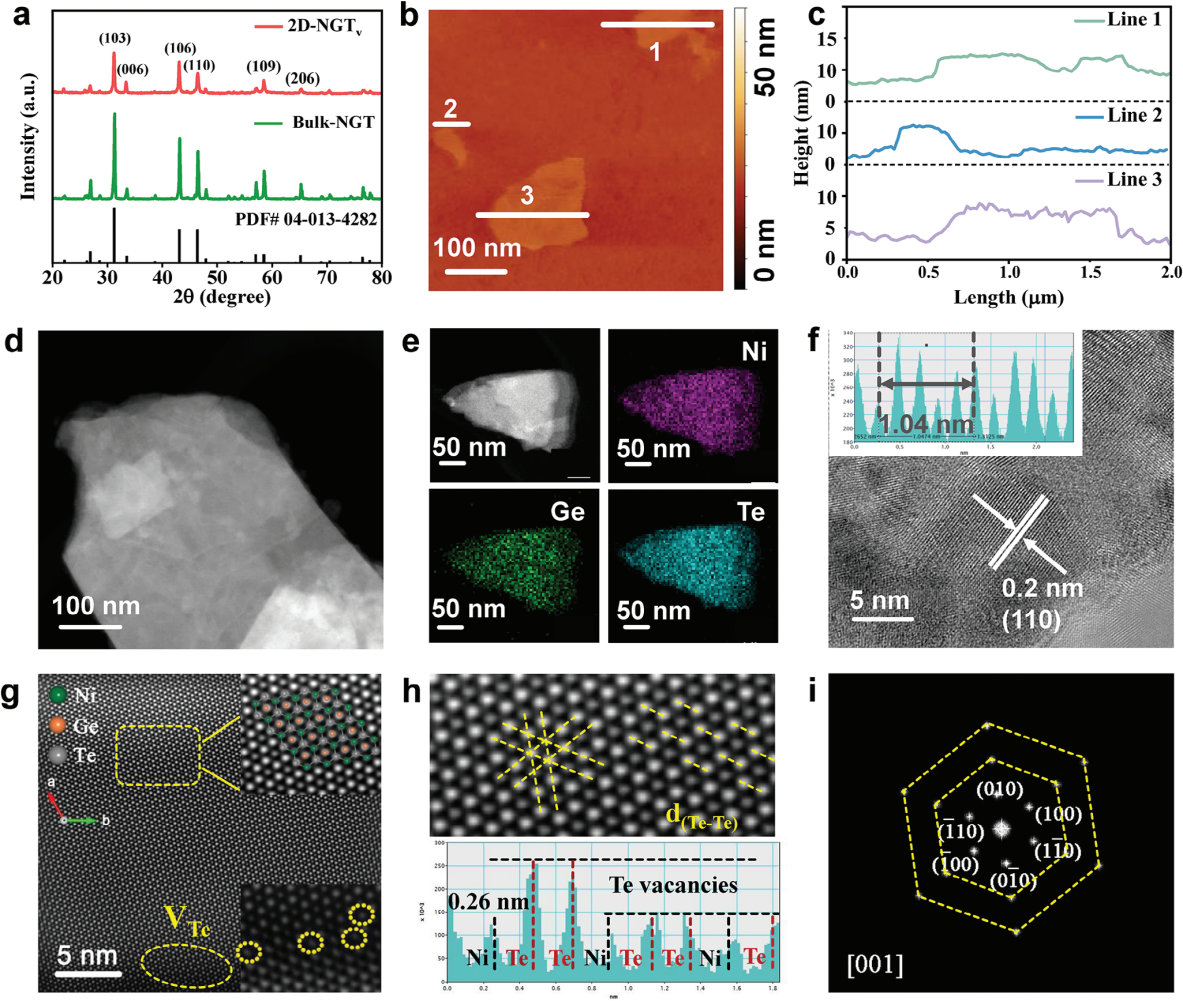
Physical characterizations of Bulk‐NGT and 2D‐NGT_v_. a) The XRD pattern of Bulk‐NGT and 2D‐NGT_v_. b,c) AFM image of 2D‐NGT_v_ and its line scan profile. d) The HAADF‐STEM image, e) the STEM‐EDS mapping, and f) the HRTEM image of 2D‐NGT_v_. g,h) HAADF‐STEM image for 2D‐NGT_v_, suggesting the presence of Te vacancies. i) The corresponding inverse FFT pattern of 2D‐NGT_v_.

Ni_3_GeTe_2_ was prepared by a solid‐state reaction^[^ ^27^ ^]^ (See Experimental Section), and the resulting Ni_3_GeTe_2_ exhibits a typical layered structure with a decent thickness and a lateral size in micrometers, which is denoted as Bulk‐NGT (Figure S1, Supporting Information). In the next step, the liquid exfoliation method was employed to synthesize thin‐layered Ni_3_GeTe_2_ by unstacking the Bulk‐NGT. The strong mechanical shearing force upon liquid exfoliation is the main driving force to overcome the relatively weak van der Waals forces of Bulk‐NGT. The well‐established liquid exfoliation strategy and our rich experience in preparing many other 2D materials have enabled us to obtain high‐quality thin‐layered Ni_3_GeTe_2_, as seen in Figures S2–S4 (Supporting Information). **Figure** **1**a displays the X‐ray diffraction (XRD) patterns of the Bulk‐NGT and thin‐layered NGT, where the diffraction peaks can be well indexed to hexagonal Ni_3_GeTe_2_ (PDF card 04‐013‐4282), indicating the successful fabrication of Ni_3_GeTe_2_. In particular, the intensity ratios of diffraction peaks (006) to (110) are 0.226 and 0.601 for bulk‐NGT and thin‐layered Ni_3_GeTe_2_, respectively, demonstrating the successful exfoliation of the structure along the *c* direction. In addition, the (006) diffraction peak is downshifted by 0.15° for thin‐layered Ni_3_GeTe_2_ in comparison to bulk‐NGT, further confirming the slight lattice expansion along the *c*‐axis deduced by layer delamination (Figure S5, Supporting Information). In Figure 1b,c, the thickness of thin‐layered Ni_3_GeTe_2_ is estimated in the range of 3 to 10 nm from the atomic force microscopy (AFM) results. Figure 1d,e presents a high‐angle annular dark‐field‐scanning transmission electron microscope (HAADF‐STEM) image of thin‐layered Ni_3_GeTe_2_ and the elemental mapping images, confirming that Ni, Ge, and Te are dispersed uniformly in the thin‐layered Ni_3_GeTe_2_ (Figure S6, Supporting Information). Moreover, the lattice plane spacing in the high‐resolution TEM image (Figure 1f) is calculated to be ≈0.2 nm, which can be indexed to the (110) plane of NGT. HAADF‐STEM images of thin‐layered Ni_3_GeTe_2_ at high magnifications are shown in Figure 1g,h and Figure S7 (Supporting Information). The atomic resolution along the [001] zone axis is presented, where the hexagonal packing of Ni, Ge, and Te atoms can be viewed. Meanwhile, the atomic stacking forms in the HAADF‐STEM image match well with the schematic atom forming deduced from the crystal structure file. In the zoomed profile in Figure 1h, the Te─Te atomic bonding can be determined, and Te atomic defects are illustrated according to the atomic Z contrast. Furthermore, compared with bulk material, the ratio of Ni to Te derived from X‐ray photoelectron spectroscopy (XPS) is increased from 1.19 for Bulk‐NGT to 1.44 for thin‐layered Ni_3_GeTe_2_, further demonstrating the formation of atomic Te defects upon exfoliation (Table S1, Supporting Information). Consequently, the thin‐layered

Ni_3_GeTe_2_ with rich atomic Te vacancies will be denoted as 2D‐NGT_v_ hereafter. Figure 1i shows the inverse FFT image processed by masking in the same area. The lattice planes of (010), (0–10), (100), (−100), (1–10), and (−110) with a respective angle of 60° are calculated and the Zone axis is determined to be [001], indexing well with the hexagon structure of NGT.^[^ ^27^ ^]^

**Figure 2:**
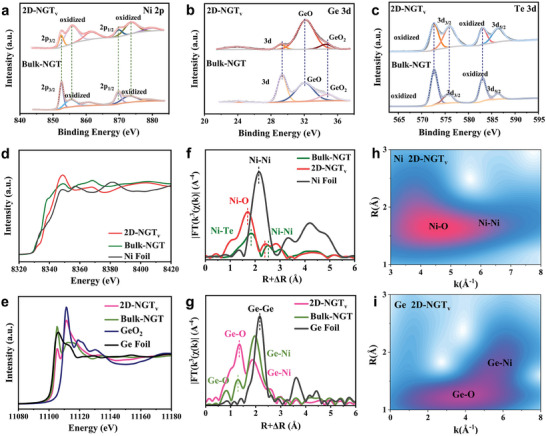
Electronic characterizations of Bulk‐NGT and 2D‐NGT_v_. a) High‐resolution XPS spectra of (a) Ni 2p spectra, b) Ge 3d spectra, c) Te 3d spectra. d) Ni K‐edge and e) Ge K‐edge XANES spectra of Bulk‐NGT, 2D‐NGT_v_, and the references. FT‐EXAFS profiles of Bulk‐NGT, 2D‐NGT_v_, and the references at f) Ni K‐edge and g) Ge K‐edge. Wavelet transform plots of h) the Ni K‐edge and i) the Ge K‐edge K^3^‐weighted EXAFS signal of 2D‐NGT_v_.

The surface chemical states and the electronic structure of the Bulk‐NGT and 2D‐NGT_v_ were analyzed by XPS. The survey spectra (Table S1, Supporting Information) confirm the presence of Ni, Ge, Te, and O for Bulk‐NGT and 2D‐NGT_v_, respectively. In **Figure** **2**a, the high‐resolution XPS Ni 2p signals for Bulk‐NGT and 2D‐NGT_v_ can be mainly deconvoluted into two components (Ni^0^ and Ni^2+^) and satellite peaks. Specifically, the binding energies (BEs) of metallic Ni doublets (Ni 2p_3/2_ and Ni 2p_1/2_) for Bulk‐NGT are located at 870.1 and 852.9 eV.^[^ ^28^ ^]^ Meanwhile, the Ni doublets at the BEs of 873.6 and 855.7 eV can be attributed to the characteristic signals of Ni^2+^.^[^ ^28^ ^]^ For 2D‐NGT_v_, the BEs of Ni^0^ and Ni^2+^ peaks remain little changed, while the peak ratio of Ni^2+^ to Ni^0^ increases significantly. The fitted results (Table S1, Supporting Information) show that the concentration of Ni^2+^ changes from 15.9% for Bulk‐NGT to 23.3% for 2D‐NGT_v_, again demonstrating that a higher proportion of Ni─O was formed for 2D‐NGT_v_ after the exfoliation process. Meanwhile, the Ge 3d spectra and the deconvoluted peaks for Bulk‐NGT and 2D‐NGT_v_ are presented in Figure 2b. The major peak located at 29.68 eV can be assigned to typical Ge^0^, and the other Ge doublets can be indexed to Ge^2+^ and Ge^4+^. Similarly, the BEs of Ge peaks show negligible shifts, but the ratio of Ge^2+^ to Ge^0^ increases from 1.6 for Bulk‐NGT to 13 for 2D‐NGT_v_, and the ratio of Ge^4+^ to Ge^0^ changed from 0.26 for Bulk‐NGT to 1.4 for 2D‐NGT_v_. The remarkably enhanced Ge─O ratio is further evidenced by the higher oxidation state of 2D‐NGT_v_ upon exfoliation. In Figure 2c, the Te 3d spectra for 2D‐NGT_v_ and Bulk‐NGT exhibit similar behavior. The much higher metal‐O ratio for 2D‐NGT_v_ in comparison to Bulk‐NGT is believed to reflect the key active site for the enhanced catalytic activity, which will be discussed thoroughly by using X‐ray absorbance spectroscopy (XAS). X‐ray absorption near‐edge structure (XANES) spectra of 2D‐NGT_v_, Bulk‐NGT, and the reference Ni foil at the Ni K‐edge are shown in Figure 2d. The Ni K‐edge scattering oscillation of 2D‐NGT_v_ is similar to that of Bulk‐NGT, but the edge position of 2D‐NGT_v_ is shifted positively compared to Bulk‐NGT, manifesting the increased average oxidation of Ni for 2D‐NGT_v_. Meanwhile, Ge K‐edge XANES profiles of the two samples and the reference Ge foil and GeO_2_ are presented in Figure 2e and Figure S8 (Supporting Information), where the white line of the Ge K‐edge has a +4.0 eV shift for 2D‐NGT_v_ in comparison to Bulk‐NGT. With Ge foil and GeO_2_ as references, the oxidation states of Ge in Bulk‐NGT and 2D‐NGT_v_ can be estimated to be ≈0.55 and ≈0.95, respectively. All these results evidence that a very high proportion of Ni─O and Ge─O moieties is formed upon the exfoliation of NGT. The improved Ni─O and Ge─O ratios for 2D‐NGT_v_ compared with Bulk‐NGT deduced from XANES profiles are consistent with the XPS results, further demonstrating the formation of electron‐deficient Ni and Ge moieties for 2D‐NGT_v_. We next employed extended X‐ray absorption fine structure (EXAFS) to elucidate the atomic coordination of 2D‐NGT_v_ versus Bulk‐NGT. Figure 2f,g, and Figures S9 and S10 (Supporting Information) show the Fourier transformed (FT) EXAFS oscillations at the Ni K‐edge and the Ge K‐edge of the as‐prepared samples in R space. For Bulk‐NGT (Figure 2f), two dominant peaks are found at the positions of 1.72 and 2.41 Å (without phase correction), which can be assigned to Ni─Te and Ni─Ni coordination, respectively. Furthermore, the coordination number of Ni─Te and Ni─Ni is calculated to be 4.8 and 9.2, respectively, based on the quantitative EXAFS fitting results (Figure S9 and Table S2, Supporting Information). In the case of 2D‐NGT_v_, two major peaks located at 1.89 and 2.51 Å are observed, which can be attributed to Ni─O and Ni─Ni interaction, respectively. Meanwhile, the fitting results demonstrate that the coordination number of Ni─O and Ni─Ni for 2D‐NGT_v_ is close to 2.5 and 3.5, respectively. The corresponding wavelet transform contour maps further demonstrate the bond transformation from Bulk‐NGT to 2D‐NGT_v_, such as the presence of Ni─O, Ni─Ni, Ge─O, and Ge─Ni in 2D‐NGT_v_ (Figure 2h,i; Figure S10, Supporting Information). Based on the aforementioned results, it can be inferred that the Ni‐Ni and Ni‐Te bonds are the two dominant bonding configurations in the Bulk‐NGT. In contrast, sufficient oxygen exposure during the layer delamination and the strong oxophilicity of Ni and Ge (since the electronegativity of O is much stronger than that of Te) leads to the generation of Ni─O and Ge─O bonds in the 2D NGT_v_. On the other hand, the O K‐edge profiles (Figure S11, Supporting Information) prove that a higher proportion of metal‐O bonding can be observed for 2D‐NGT_v_ compared with Bulk‐NGT, again demonstrating the generation of the electron‐deficient metal‐O species. Consequently, Ni‐Ni bonds remain slightly changed, while Ni─O bonds increase significantly for 2D‐NGT_v_. For the same reason, the coordination number of Ge─O changes from 4.6 for bulk‐NGT to 1.7 for 2D‐NGT_v_, while the coordination number of Ge─Ni is slightly decreased from Bulk‐NGT to 2D‐NGT_v_. Perfect agreement between the XPS results and the XAS profiles can be found, again manifesting that the highly improved metal‐O coordination is probably the main reason for the highly accelerated oxygen evolution kinetics.

**Figure 3:**
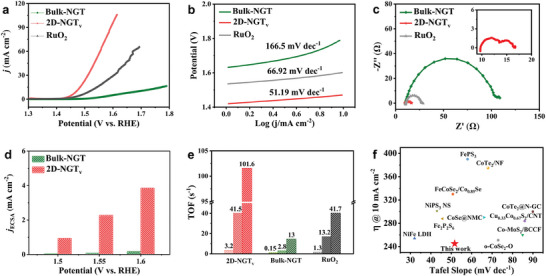
Electrochemical characterizations of Bulk‐NGT and 2D‐NGT_v_. a) LSV curves, b) the Tafel plots, and c) the EIS spectra (at 1.65 V vs RHE) of Bulk‐NGT, 2D‐NGT_v_, and the references. d) ECSA normalized OER activity and e) TOF values (at the overpotentials of 200, 300, and 400 mV) of Bulk‐NGT, 2D‐NGT_v_, and RuO_2_, respectively. f) OER performance comparison between 2D‐NGT_v_ and the reported OER electrocatalysts at the current density of 10 mA cm^−2^.

The electrocatalytic oxygen evolution activity of Bulk‐NGT and 2D‐NGT_v_ was evaluated in 1 m KOH solutions using the rotating disk electrode configuration. The calibration of the reference electrode was conducted in the 1 m Ni KOH solution (Figure S12, Supporting Information). Linear sweep voltammetry (LSV) curves of the as‐prepared catalysts are presented in **Figure** **3**a and Figures S13 and S14 (Supporting Information). It shows that 2D‐NGT_v_ exhibits a remarkably higher current density compared with the benchmark RuO_2_, not even to mention Bulk‐NGT. The overpotential at the current density of 10 mA cm^−2^ for 2D‐NGT_v_ is 250 mV, much lower than the 300 mV for RuO_2_ and the 470 mV for Bulk‐NGT. Impressively, to drive the current density of 100 mA cm^−2^, 2D‐NGT_v_ only needs 370 mV as overpotential, which is considerably lower than many typical 2D catalysts, such as Ni─Mo─S with 390 mV.^[^ ^29^, ^30^ ^]^ Moreover, we further employed mass‐normalized activity to justify the activity of the as‐prepared catalysts (Figure S15, Supporting Information). Notably, the mass‐normalized current density for 2D‐NGT_v_ at the overpotential of 250 mV reaches 391 mA mg^−1^, which is >3 times higher than that for RuO_2_ (111 mA mg^−1^) and 30 times higher than Bulk‐NGT (12.4 mA mg^−1^), respectively. Detailed reaction kinetics was analyzed through the Tafel plot (Figure 3b). 2D‐NGT_v_ is found to have the lowest Tafel plot compared with RuO_2_ and Bulk‐NGT, again manifesting its faster charge transfer kinetics. The faster charge transfer kinetics of 2D‐NGT_v_ among these catalysts can be further evidenced by the electrochemical impedance spectroscopy (EIS) spectra, as shown in Figure 3c. From the Nyquist plot, the 2D‐NGT_v_ displays the lowest charge transfer resistance (16.82 Ω) than the Bulk‐NGT (108.61 Ω) and RuO_2_ (28.07 Ω) at the overpotential of 1.65 V versus RHE. The equivalent circuit and the fitted results are shown in Figure S16 and Table S3 (Supporting Information), again confirming the enhanced OER transfer kinetics for 2D‐NGT_v_. To further evaluate the intrinsic activity of 2D‐NGT_v_ and Bulk‐NGT, the electrochemically active surface area (ECSA) was assessed by cyclic voltammetry (CV) (Figure S17, Supporting Information), and the ECSA normalized current density of the as‐prepared catalysts is shown in Figure 3d. On the one hand, the slightly increased pseudo capacitance of 2D‐NGT_v_ (0.228 mF cm^−2^) in comparison to Bulk‐NGT (0.196 mF cm^−2^) implies that the exfoliation strategy promotes the increased exposure (≈1.2 times higher) of active sites for 2D‐NGT_v_. On the other hand, the ECSA normalized current density shows that the current density of 2D‐NGT_v_ approaches 3.85 mA cm^−2^ at the potential of 1.6 V, which is 22.6 times higher than that of Bulk‐NGT (0.17 mA cm^−2^). Such a considerably improved ECSA normalized current density indicates that the outstanding OER activity is not merely attributed to the increased active sites but to the vastly enhanced intrinsic activity. Based on the discussions of the XAS and XPS sections, it is reasonable to conclude that the in‐situ formed Ni─O and Ge─O moieties and rich Te vacancies are critical active sites for the primarily enhanced intrinsic activity. In addition, the turnover frequency (TOF) of RuO_2_, Bulk‐NGT, and 2D‐NGT_v_ is presented in Figure 3e, where the considerably enhanced TOF value for 2D‐NGT_v_ again validates the highly improved intrinsic OER activity. Compared with typical 2D materials such as MoS_2_, FePS_3_, and even NiFe LDH, the as‐prepared 2D‐NGT_v_ still exhibits competitive alkaline OER performance^[^ ^31^, ^32^, ^33^, ^34^, ^35^, ^36^, ^37^, ^38^, ^39^, ^40^, ^41^ ^]^ (Figure 3f; Table S4, Supporting Information). Moreover, the XRD patterns and the TEM images 2D‐NGT_v_ after the course of OER are shown in Figures S18 and S19 (Supporting Information), where the strong intensity peak at ≈26° ≈40.0° and ≈54.5° can be ascribed to the carbon paper (Toray TGP‐H‐090). Meanwhile, the peaks at the 2 theta degree of 31.41°, 33.73°, 43.34°, and 46.69° can be indexed to (103), (006), (106), (110) planes of 2D‐NGT_v_, confirming the presence of NGT even after the electrolysis reactions. These results validate that the NGT structure can be sustained after the course of OER. On the other hand, the TEM, HRTEM, and HAADF‐STEM images of 2D‐NGT_v_ are depicted in Figure S19 (Supporting Information). The diffraction pattern is displayed in Figure S19b (Supporting Information), where the lattice planes of (−1−10), (2–10), (1–20), (110), (2–10), and (1–20) with a respective angle of 60° along the Zone axis of [001] are determined. Meanwhile, the d‐spacings of the lattices in Figure S19c,d (Supporting Information) are calculated to be ≈0.2 nm, aligning well with the (110) lattice plane of NGT. The XRD patterns and the TEM results collectively demonstrate that the NGT structure is well sustained after OER conditions.

**Figure 4:**
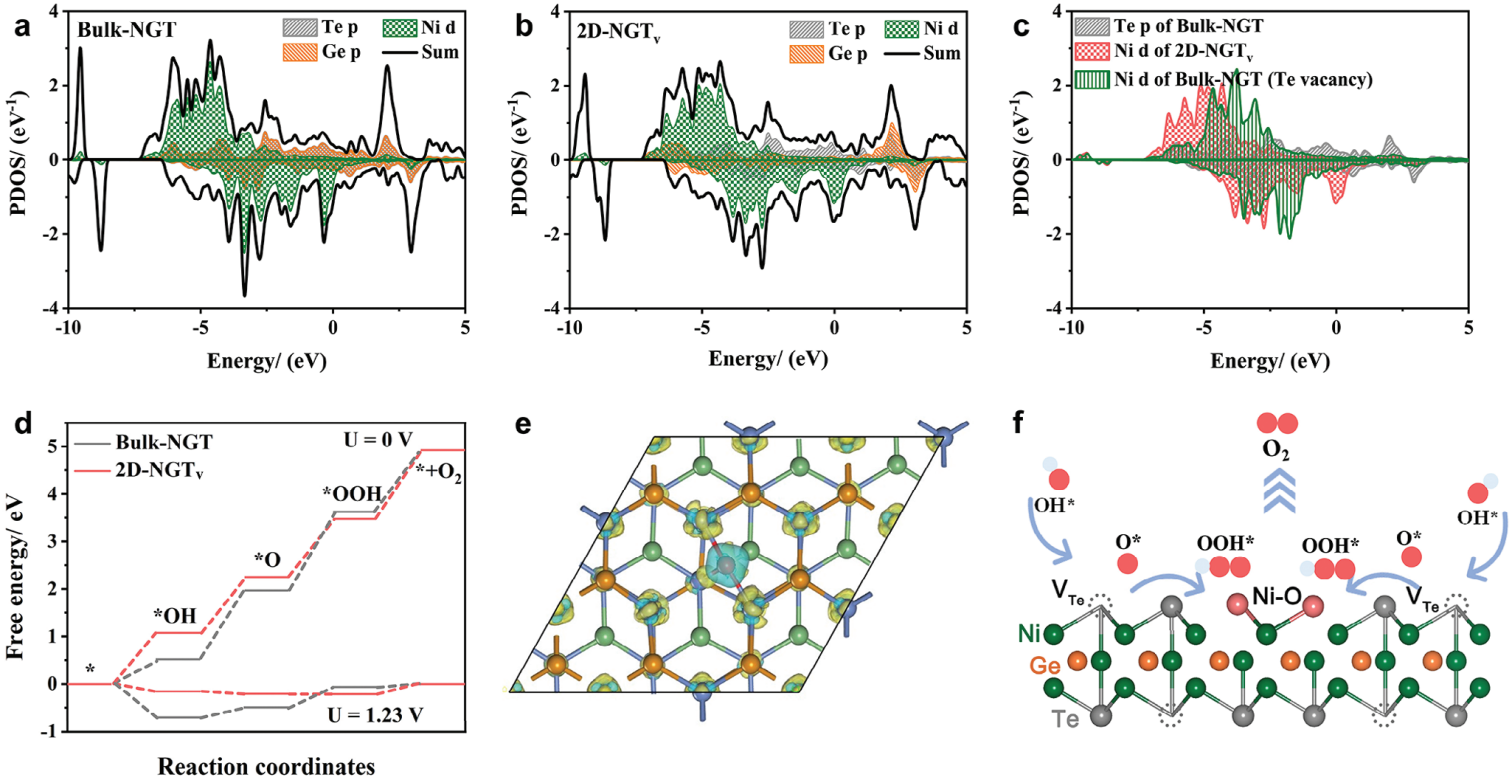
Theoretical calculation of OER mechanisms on Bulk‐NGT and 2D‐NGT_v_. PDOS of Ni d, Ge p, and Te p orbitals for a) Bulk‐NGT, b) 2D‐NGT_v_. c) Bulk‐NGT with Te vacancies. d) Free energy diagram of the OER for Bulk‐NGT and 2D‐NGT_v_. e) Charge density difference of 2D‐NGT_v_. f) Schematic image of the OER mechanisms for 2D‐NGT_v_.

To further clarify the distinct OER reaction kinetics on the 2D‐NGT_v_ versus Bulk‐NGT, we employed DFT calculations to analyze the electronic structures of the as‐prepared NGT samples and the probable reaction mechanisms. As shown in **Figures** **4**a–c and S20 (Supporting Information), the calculated project density of states (PDOS) of bulk‐NGT and 2D‐NGT_v_ are presented. For bulk‐NGT, a significantly higher population of Ni 3d state and Ge p state is found at the Fermi level, indicating the metallic states of the bulk‐NGT samples. For comparison, we built a 2D‐NGT_v_ model by creating Te vacancies and surface O species. Notably, a considerable amount of Ni d states are found crossing the Fermi level while no obvious Te p states can be observed. This result is in agreement with previous theoretical reports. It again demonstrates that Te vacancies and surface O moieties do not affect the metallic features of 2D‐NGT_v_, which is also a key factor for the much‐enhanced OER reaction kinetics. A detailed PDOS analysis between Bulk‐NGT, 2D‐NGT_v_, and NGT‐bearing Te vacancy is shown in Figure 4c. Furthermore, the Gibbs free energy of the four elementary steps on Bulk‐NGT, NGT (Te vacancy), and 2D‐NGT_v_ is depicted in Figure 4d and Figure S21 (Supporting Information). Specifically, the rate‐determining step (RDS) for Bulk‐NGT is determined to be the adsorption of *O and the formation of *OOH, due to the very low Gibbs free energy for adsorbing *O and the high binding strength. Meanwhile, the incorporation of Te vacancies does not change the reaction barrier too much, and the RDS for NGT (Te vacancy) is maintained to be the combination of *O and hydroxyl for generating *OOH. The two models indicate that the basal NGT plane and NGT plane with Te vacancies are not effective for boosting the formation of *OOH, thereby resulting in considerably high reaction overpotential. Notably, for 2D‐NGT_v_, the rich metal‐O moieties endow Ni/Ge 3d state with much higher overlap with O 2p orbital, thus contributing to the facile adsorption of oxygen species as well as improved charge transfer kinetics. Consequently, the reaction overpotential is calculated to reduce from 0.429 V for NGT to 0.217 V for 2D‐NGT_v_, respectively. The calculated differential charge density distribution is shown in Figure 4e, where the electron transfer between Ni and O is observed. This further consolidates our consumption that the boosted metal species are critical for activating intermediate adsorption and accelerating the charge transfer kinetics. A schematic reaction mechanism is presented in Figure 4f. Compared to the basal NGT plane, 2D‐NGT_v_ has enhanced intrinsic activity stemming from both the Te vacancies and metal‐O moieties. The Te vacancies are active for initial *OH adsorption and the formation of *O, as demonstrated by our calculation results and previous reports. Meanwhile, the metal‐O species are dedicated to boosting the subsequent generation of *OOH and O_2_ formation owing to their significantly enhanced Ni d‐O p orbital hybridization, thus contributing synergistically to the overall fast oxygen evolution kinetics. To analyze the surface chemical states of Bulk‐NGT and 2D‐NGT_v_, the XPS spectra of Ni 2p, Ge 3d, Te 3d, and O 1s for Bulk‐NGT and 2D‐NGT_v_ tested with a series of etching times (from 0 s to 180 s) further demonstrate the gradually enhanced oxygen content (Figures S22 and S23, Supporting Information), again manifesting a higher proportion of metal‐O moieties is a necessity for the improved OER activity.

**Figure 5:**
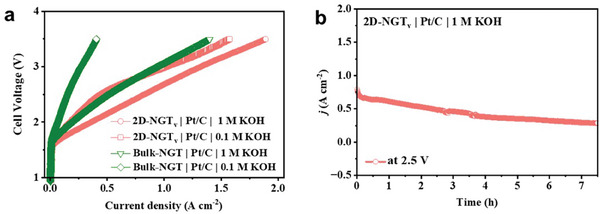
a) LSV curves of Bulk‐NGT and 2D‐NGT_v_‐based AEM water electrolyzers feeding with 1 m KOH and 0.1 m KOH. b) The stability test of the AEM electrolyzer at the voltage of 2.5 V for 7.5 h.

**Figure 6:**
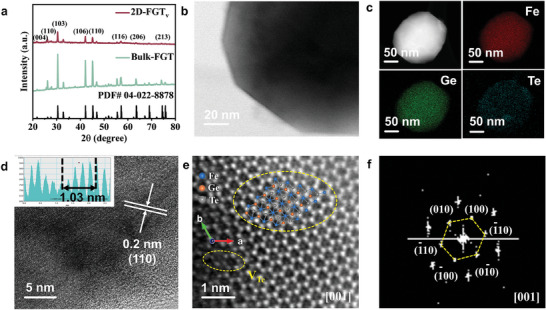
a) XRD patterns of Bulk‐FGT and 2D‐FGT_v_. b) TEM image, c) STEM‐EDS mapping image, d) HRTEM image, e) HAADF‐STEM image, and corresponding FFT pattern of 2D‐FGT_v_.

The research on the AEM water electrolyzer was conducted and the results are presented in **Figure** **5**. Specifically, we employed Bulk‐NGT versus 2D‐NGT_v_ as the cathode catalyst and 20% Pt/C (commercial sample from Aldrich) as the anode catalyst, respectively to assemble the catalyst‐coated membrane. Anion‐exchange membrane (Fumasep FAA‐3‐50, Fuel Cell Store) was used as the anion‐exchange separator and what we did was to coat anode/cathode catalyst inks onto carbon paper.^[^ ^42^, ^43^ ^]^ In the next step, the anion‐exchange membrane water electrolyzer was assembled by placing endplates, gaskets, O‐rings, and the catalysts‐coated membrane in the right sequence and the two types of feeding electrolytes (1 m KOH solution vs 0.1 m KOH solution) were employed^[^ ^5^, ^44^ ^]^ (Figure S24, Supporting Information). As shown in Figure 5a, when changing the electrolyte from 0.1 m KOH solution to 1 m KOH solution, both Bulk‐NGT and 2D‐NGT_v_ deliver remarkably enhanced current density at the identical potential. This is because, at 1 m KOH solution, the highly improved ion conductivity favors the reaction kinetics.^[^ ^45^, ^46^ ^]^ For a 2D‐NGT_v_‐based AEM device, the current density reaches 1.02 A cm^−2^ at the voltage of 3 V in 0.1 m KOH solution and this value further increases to 1.32 A cm^−2^ in 1 m KOH solution. The AEM water electrolyzer was tested under a fixed potential (2.5 V) to evaluate the device's stability under working conditions. As depicted in Figure 5b, the cell exhibits decent device stability within 7.5 h of operation and this implies that 2D‐NGT_v_ delivers nice overall stability even in a practical AEM water electrolyzer. These results confirm that it is a promising candidate for potential applications as an OER electrocatalyst. Similar to NGT, Bulk‐Fe_3_GeTe_2_ (denoted as FGT) and 2D‐FGT_v_ were synthesized, following the identical preparation strategy, respectively. XRD patterns (**Figure** **6**a; Figure S25, Supporting Information) and the intensity ratio of the (004) plane to (110) confirm that the 2D‐FGT_v_ is successfully exfoliated along the c direction (PDF card 04‐022‐8878). Furthermore, the downshift of (004) diffraction peak of 0.05° indicates the lattice expansion after exfoliation to layered FGT. In Figure 6b–d and Figures S26–S28 (Supporting Information), plane spacing is nearly 0.2 nm, corresponding to the (110) plane. HAADF‐STEM demonstrates the hexagonal atomic stacking of Fe, Ge, and Te along the c‐axis (Figure 6e,f; Figure S29, Supporting Information). The electrocatalytic OER performances of 2D‐FGT_v_ and Bulk‐FGT are presented in Figure S30 (Supporting Information). Specifically, the much‐improved OER overpotential and reaction kinetics for 2D‐FGT_v_ compared with bulk‐FGT further suggests that 2D ferromagnetic M_3_GeTe_2_ (M = Ni and Fe) are efficient electrocatalysts for oxygen evolution reactions.

## 3 Conclusion

In summary, ferromagnetic material M_3_GeTe_2_ (M = Ni/Fe, denoted as MGT) was experimentally prepared as 2D nanosheets bearing rich atomic vacancies (2D‐MGT_v_) via a general solution‐based exfoliation method and demonstrated as an efficient electrocatalyst toward alkaline oxygen evolution reaction. The in‐situ generated atomic vacancies and surface metal‐oxygen configuration along exfoliation endow 2D‐MGT_v_ with considerably enhanced intrinsic activity toward electrocatalytic reactions, which is a distinctive viewpoint compared with previous works that were studied by theoretical simulations. Consequently, the 2D‐MGT_v_ were found to have highly improved reaction kinetics owing to the synergistically promoted adsorption toward key intermediates of OH*, O*, and OOH* and the optimized charge transfer kinetics, as confirmed by experimental analysis and DFT calculations. Our work is expected to shed light on the rational design and preparation of 2D ferromagnetic materials as efficient electrocatalysts for a wider range of electrocatalytic applications.

## 4 Experimental Section

### Preparation of Bulk‐MGT and 2D‐MGT_v_ materials

Bulk‐MGT was prepared via a solid‐state sintering method. Iron (99.99%), germanium (99.99%), tellurium (99.99%), and nickel (99.99%), germanium (99.99%), tellurium (99.99%) with calculated amounts were enclosed in a quartz glass ampoule. The sealed ampoules were sintered in a muffle furnace at 700 °C for 10 days. After that, the samples were cooled to room temperature. Bulk‐MGT was obtained and stored in the glove box under an Argon atmosphere.

2D‐MGT_v_ was obtained by shear force exfoliation. Bulk‐MGT (2 g) was added to 150 mL dimethyl formamide (DMF) solution, and the material was exfoliated by shear force exfoliation for 2 h at 5000 rpm in DMF solution (Silverson L5M). Ice cubes were supplied during the exfoliation process. After exfoliation, the as‐prepared suspension was centrifuged at 5000 rpm for 15 min to remove the large bulk material. The suspension was washed and then dried at 60 °C, Finally, 2D‐MGT_v_ was collected for the tests.

### Material Characterizations

XRD patterns were collected using Cu Kα radiation (*λ* = 1.541Å). XPS spectra were characterized by a Phoibos 100 Analyzer (SPECS, Germany, Al Kα X‐rays). TEM images were obtained with a JEOL JEM F200 microscope operating at a working voltage of 200 kV. Scanning transmission electron microscopy ‒ energy dispersive X‐ray spectroscopy (STEM‐EDS) images were obtained on a JEOL ARM200F at 200 kV. The Near‐edge X‐ray fine structure (NEXAFS) characterization for O K‐edge was processed by soft X‐ray beamline. X‐ray absorption (XAS) studies were used to analyze the local chemical and coordination environment for Ni, Ge, and Te. Ni, Ge, and Te K‐edge spectra were collected on a beamline at the Australian Synchrotron. The data was processed using Demeter software. The wavelet transformation was performed using the Igor Pro script by Funke et al. This procedure was focused on characterizing the backscattering atoms and the bond atoms and bond lengths.

### DFT Calculations

FeGeTe(001), FeGeTe(001)‐V_Te_, and FeGeTe(001)‐V_Te_+O surfaces were constructed, and then the intermediates of OH, O, and OOH groups were absorbed on the Te and Ni sites. All atoms were allowed to relax to the minimum in their enthalpy without any constraints. First‐principles calculations in the framework of density functional theory, including structural and electronic performances, were carried out based on the Cambridge Sequential Total Energy Package known as CASTEP.^[^ ^47^ ^]^ The exchange‐correlation function under the generalized gradient approximation (GGA) with norm‐conserving pseudopotentials and the Perdew–Burke–Ernzerhof applicable was adopted to describe the electron–electron interaction. An energy cut‐off of 750 eV was used, and a k‐point sampling set of 7 × 7 × 1 was tested for convergence. A force tolerance of 0.01 eV Å^−1^, energy tolerance of 5.0 × 10^−7^ eV per atom, and maximum displacement of 5.0 × 10^−4^ Å were considered. The GGA+U approximation, where *U* is the Hubbard correction, with spin polarization, was adopted to describe the localized *d* states, where *U* = 3.29 eV was applied for the Ni atom. The Grimme method for DFT‐D correction was considered for all calculations.^[^ ^48^, ^49^ ^]^

### Thermodynamics

The adsorption energy Δ*E* for *A* = OH, O, and OOH groups on the surfaces of substrates was defined as:^[^ ^50^ ^]^

(1) $$\Delta E\;=E_{*A}\;-\left(E_{*}+E_{A\;}\right)$$

where *A and * denote the adsorption of A groups on substrates and the bare substrates, while *E*_A_ denotes the energy of A groups.

The free energy change Δ*G* of the reaction was calculated as the difference between the free energies of the initial and final states, as shown below:^[^ ^50^ ^]^

(2) ΔG=ΔE+ΔZPE−TΔS

where ∆*E* is the energy change between the reactant and product obtained from DFT calculations; ∆*ZPE* is the change in the zero‐point energy; *T* and *∆S* denote the temperature and the change of entropy, respectively. Herein, *T* = 300 K was considered.

The electrochemical model of the oxygen evolution reaction/ oxygen reduction reaction (OER/ORR) in alkaline media could be divided into four one‐electron reactions:

(3) $$*+OH^{-}↔OH^{*}+e^{-}$$

(4) $$OH^{*}+OH^{-}↔O^{*}+H_{2}O\;\left(l\right)+e^{-}$$

(5) $$O^{*}+OH^{-}↔OOH^{*}+\;e^{-}$$

(6) $$OOH^{*}+OH^{-}↔\;*+O_{2}\left(g\right)+H_{2}O\left(l\right)+e^{-}$$

where the * denotes the active site. The adsorption energies of intermediates (OH, O, and OOH groups) on substrates were calculated by the following equations:

(7) $$\Delta \;E_{*O}=\;E\left(sub/O\right)-E\left(sub\right)-\left[E(H_{2}O\right)-E\left(H_{2}\right)]]$$

(8) $$\Delta \;E_{*OH}=\;E\left(sub/OH\right)-E\left(sub\right)-\left[E(H_{2}O\right)-E\left(H_{2}\right)/2]$$

(9) $$\Delta \;E_{*OOH}=\;E\left(sub/OOH\right)-E\left(sub\right)-\left[2\times E(H_{2}O\right)-3\times E\left(H_{2}\right)/2]$$

where *E*(sub/H_2_O), *E*(sub/OH), *E*(sub/O) and *E*(sub/OOH) denoted the total energies of H_2_O, OH, O, and OOH groups on substrates. *E*(sub), *E*(H_2_O), and *E*(H_2_) were the energies of the bare substrate, water, and hydrogen gas, respectively.

The Gibbs free energy changes of Equations ((3), (4), (5), (6)) could be estimated by:

(10) $$\Delta \;G_{1}=\;\Delta G_{OH*}$$

(11) $$\Delta \;G_{2}=\;\Delta G_{O*}-\Delta G_{OH*}$$

(12) $$\Delta \;G_{3}=\;\Delta G_{OOH*}-\Delta G_{O*}$$

(13) $$\Delta \;G_{4}=\;4.92eV-\Delta G_{OOH*}$$

where the sum of ΔG_1‐4_ was fixed to the negative of the experimental Gibbs free energy of the formation of two water molecules ($-2\;\Delta_{H_{2}O}^{exp}=\;4.92\;eV)$. The Gibbs free energy of (H^+^ + e^−^) in solution was estimated as half the energy of the H_2_ molecule in the standard condition.

The overpotential of the OER was determined by the following Equations:

(14) $$\eta^{OER}=U_{OER}\;-1.23$$

(15) $$U_{OER}=\;Max\;\left(\Delta G_{1},\Delta G_{2},\;\Delta G_{3},\Delta G_{4}\right)/e$$

### Electrochemical Measurements

All electrochemical data were collected by a VSP 300 electrochemical workstation. A rotating disk electrode and WaveDriver system (Pine Instruments) were employed. All measurements were conducted in 1 m KOH (potassium hydroxide (90%, reagent grade, flakes)) solution. Hg/HgO was used as the reference electrode, and a graphite rod was employed as the counter electrode. The catalyst ink that was deposited on the glassy carbon electrode with 0.196 cm^−2^ of the effective working area was used as the working electrode. To prepare the working electrodes, 2 mg electrocatalyst was dispersed in Nafion mixed solution, which contained 16 µL Nafion solution (Nafion 117, 5%), 384 µL deionized water, and 100 µL isopropanol, for ultrasonication for 2 h to obtain a uniform ink. Then, 10 µL ink was dropped on the polished glassy carbon electrode and dried in air. The working electrode was rotated at 1600 rpm during measurements. CV was conducted from 1.02 to 1.72 V versus RHE at the scan rate of 10 mV s^−1^ before LSV testing. The LSV curves were recorded at 5 mV s^−1^. All LSV curves were corrected with 95% *iR* compensation. EIS was conducted at 1.65 V versus RHE in the frequency range of 10–100 kHz. The ECSA was estimated based on the following Equations:

(16) $$iC\;=\;VC_{DL}$$

(17) $$ECSA\;=C_{DL}\;/C_{S}$$

The ECSA was calculated with double‐layer capacitance (*C*_DL_) and specific capacitance (*C*_S_) in 1 m KOH.

### Electrochemical Measurements in AEM Electrolyzer

The AEM testing follows the recent procedure, initiating with the immersion of the ion‐exchange‐memberane into KOH solution for 24 h before constructing the AEM electrolyzer. Bulk‐NGT and 2D‐NGT_v_ were employed as anode catalysts, separately. 20% Pt/C (Sigma‐Aldrich) was used as a cathode catalyst. The AEM electrolyzer was assessed at 20 °C, using 1.0 m KOH and 0.1 m KOH with a flow rate of ≈40 mL mi^−1^n, respectively. Prior to AEM testing, 10 cycles of CV were performed at a scan rate of 100 mV s^−1^. Subsequently, the AEM electrolyzer was operated at 2 V for 5 min for stabilization. For stability measurement, the catalyst ink was prepared as described earlier, and 20% Pt/C and 2D‐NGT_v_ served as cathode and anode, separately, with an active surface area of 1 × 1 cm^−2^. The CA test was conducted at 20 °C, using 1.0 m KOH as electrolyte with a flow rate of 40 mL min^−1^.

## Conflict of Interest

The authors declare no conflict of interest.

## Supporting information

Supporting Information

## Data Availability

The data that support the findings of this study are available from the corresponding author upon reasonable request.
