# Supplementary material for: 2D Ferromagnetic M3GeTe2 (M = Ni/Fe) for Boosting Intermediates Adsorption toward Faster Water Oxidation
Source: Adv Sci (Weinh). 2024 Mar 16;11(21):2310115. doi: 10.1002/advs.202310115 (PMC11151052; doi:10.1002/advs.202310115)
Supplement: Supplementary file 1 — Supporting Information [file ADVS-11-2310115-s001.pdf]

ADVANCED SCIENCE
Open Access

Supporting Information

for *Adv. Sci.*, DOI 10.1002/advs.202310115

2D Ferromagnetic M_3GeTe_2 (M = Ni/Fe) for Boosting Intermediates Adsorption toward Faster Water Oxidation

Guyue Bo, Peng Li, Yameng Fan, Xiaobo Zheng, Mengting Zhao, Qiang Zhu, Yang Fu, Yitong Li, Wei Kong Pang*, Wei Hong Lai, Bernt Johannessen, Lars Thomsen, Bruce Cowie, Tianyi Ma, Cheng Wang, Guan Heng Yeoh, Yi Du, Shi Xue Dou and Xun Xu**

Supporting Information

2D Ferromagnetic M_3GeTe_2 (M = Ni/Fe) for Boosting Hydroxyl Adsorption toward Highly Efficient Water Oxidation Reaction

Guyue Bo,[a] Peng Li,[b] Yameng Fan,[a] Xiaobo Zheng,[a] Mengting Zhao,[c] Qiang Zhu,[d] Yitong Li,[b] Wei Kong Pang,[a*] Wei Hong Lai,[a] Bernt Johannessen,[a,e] Lars Thomsen,[e] Bruce Cowie,[e] Cheng Wang,[f] Guan Heng Yeoh,[f] Yi Du,[g] Shi Xue Dou,[a,h] Xun Xu[a*]*

[a] Institute for Superconducting & Electronic Materials, Australian Institute for Innovative Materials, University of Wollongong, Wollongong, NSW 2522, Australia

[b] School of Science, RMIT University, Melbourne, VIC 3000, Australia

[c] School of Physics and Astronomy, Monash University, Clayton, VIC 3800, Australia

[d] Electron Microscopy Center, University of Wollongong, Wollongong, NSW 2500, Australia

[e] Australian Synchrotron, Australian Nuclear Science and Technology Organization Clayton, Victoria 3168, Australia

[f] School of Mechanical and Manufacturing Engineering, University of New South Wales, 2052, Australia

g School of Physics and BUAA-UOW Joint Research Centre, Beihang University, Beijing 100191, P.R. China

[h] Institute of Energy Materials Science (IEMS), University of Shanghai for Science and Technology, 516 Jungong Road, Shanghai, 200093, China

*Correspondence to: Peng Li (peng.li2@rmit.edu.au) (P.L.), Wei Kong Pang (wkpang@uow.edu.au) (W.K.P.), and Xun Xu (xun@uow.edu.au) (X.X.)

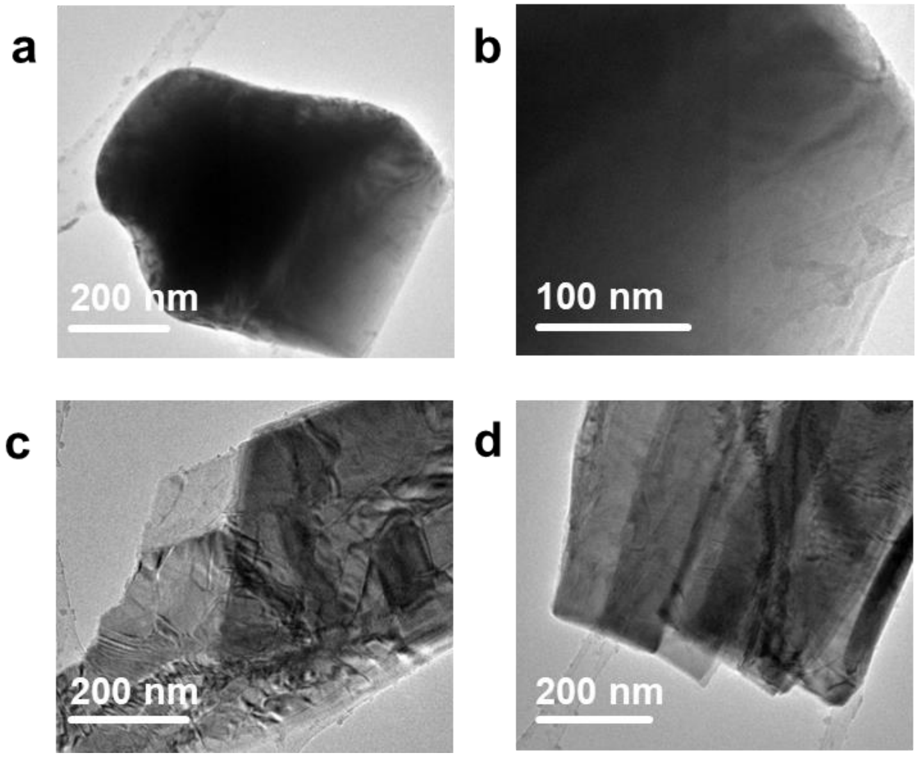

Figure S1. TEM images of Bulk-NGT.

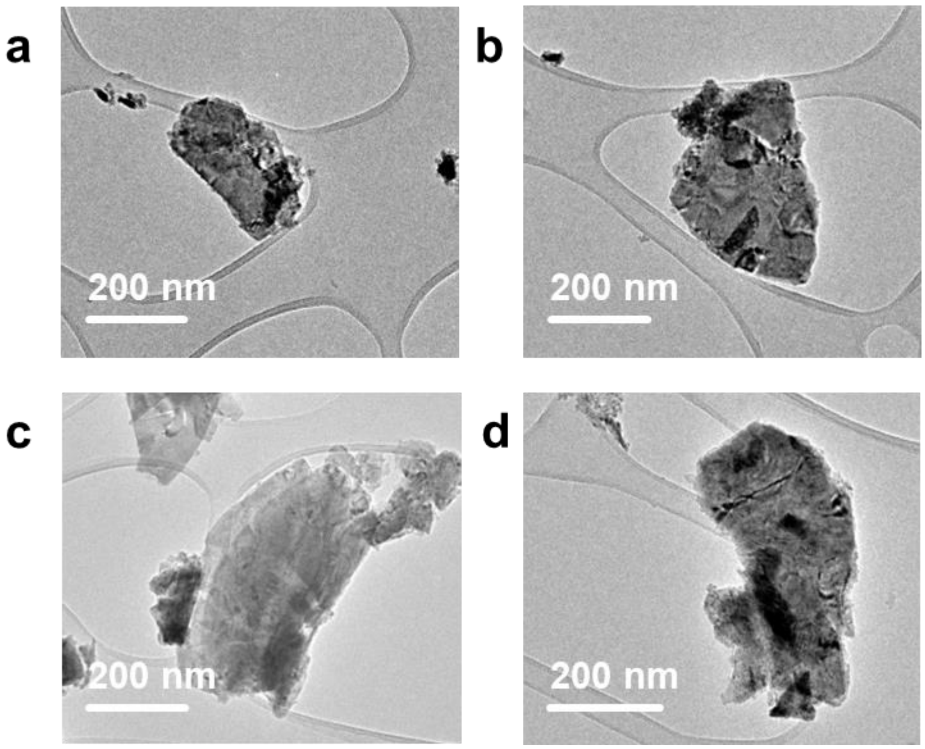

Figure S2. TEM images of 2D-NGT$_v$.

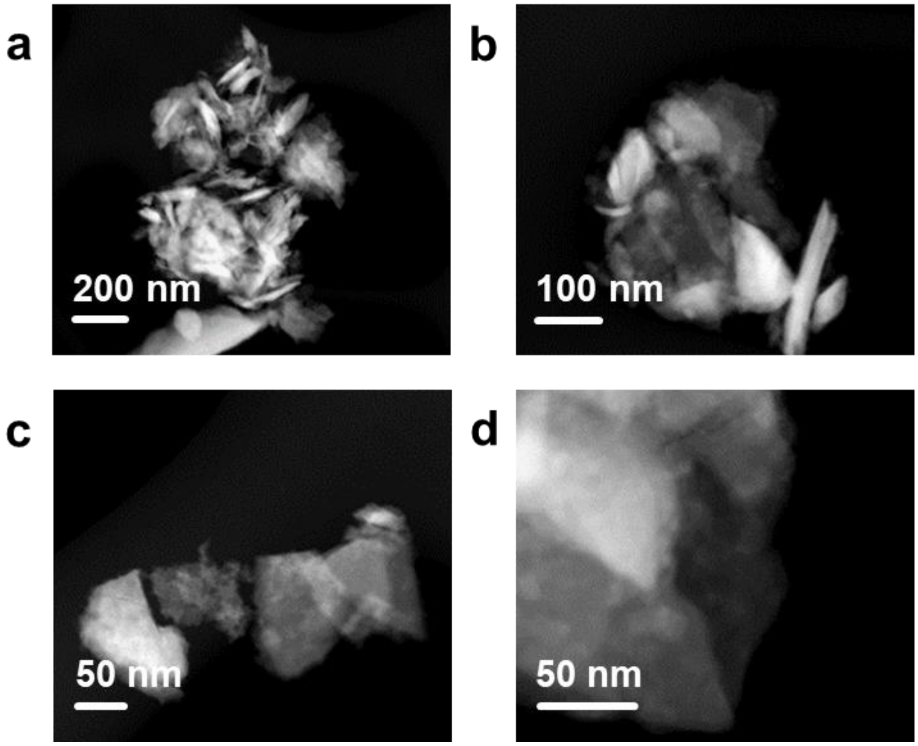

Figure S3. HAADF-STEM images of 2D-NGT_v.

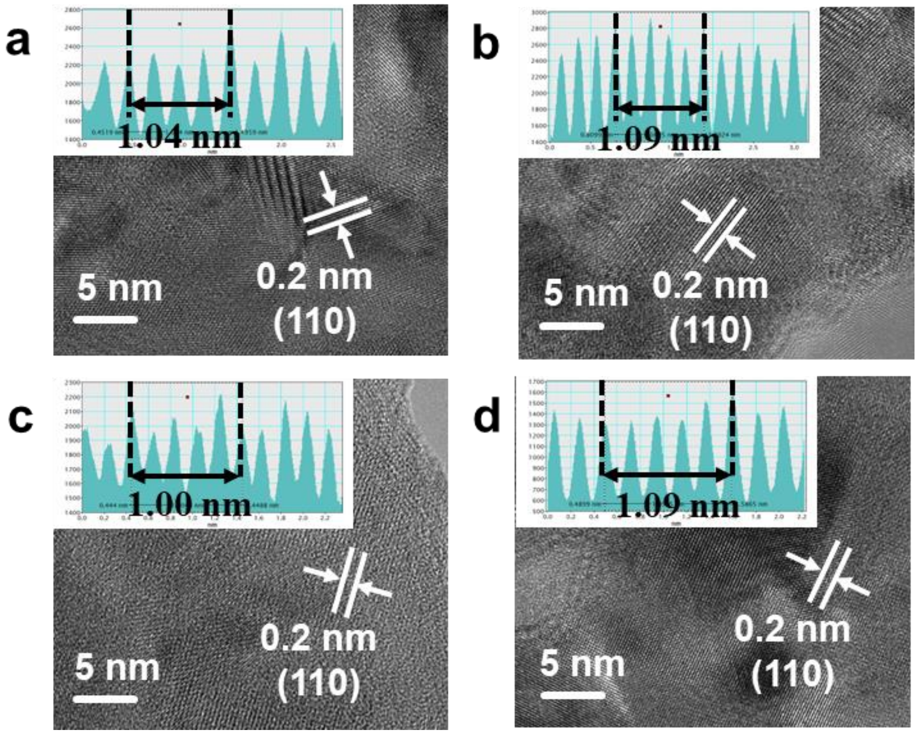

Figure S4. HRTEM Images of 2D-NGT_V.

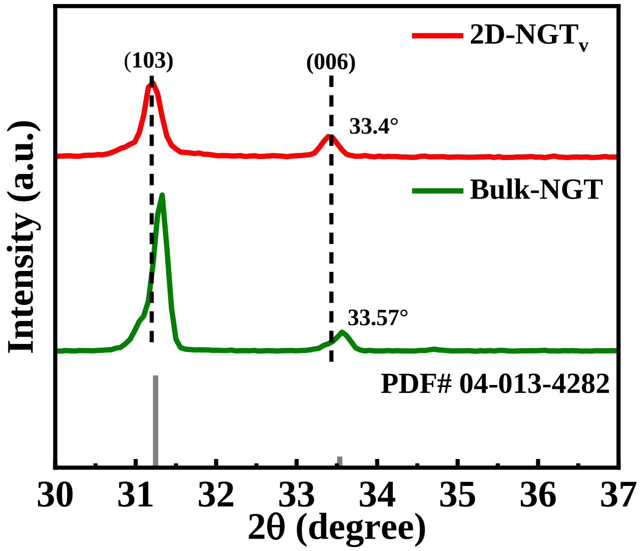

Figure S5. X-ray diffraction (XRD) patterns of Bulk-NGT and 2D-NGT$_v$ (30° - 37°). The details about the diffraction peak of (006) for Bulk-NGT and 2D-NGT$_v$ indicate the lattice expansion.

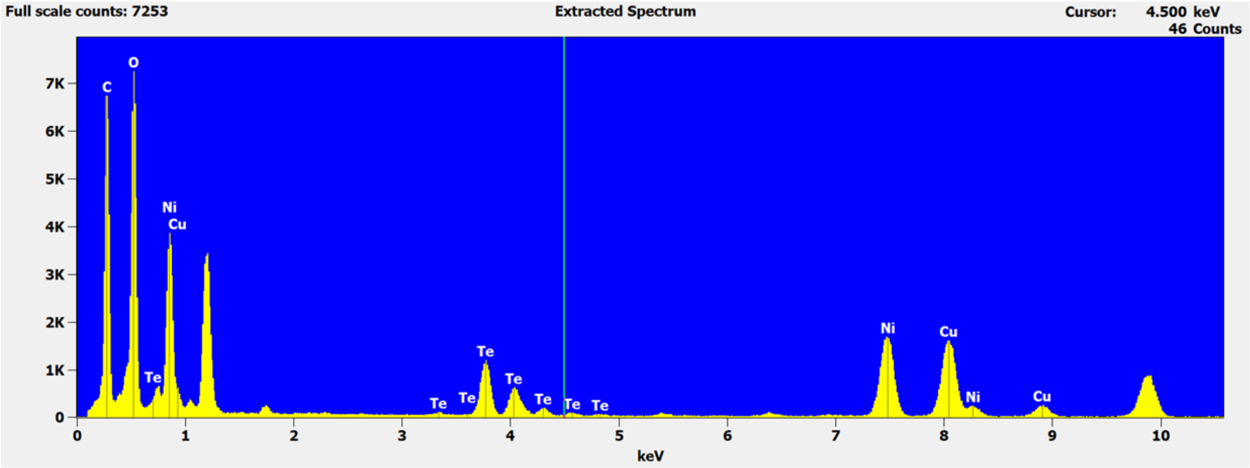

Figure S6. EDS spectrum of 2D-NGT$_v$, confirming the existence of Ni, Ge, and Te in the sample.

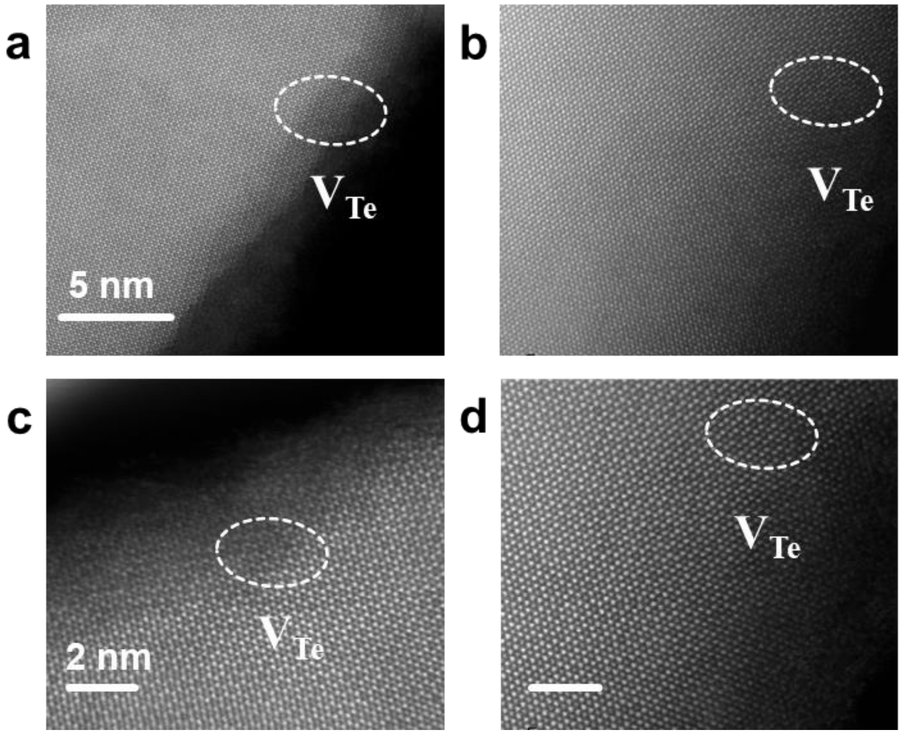

Figure S7. STEM of 2D-NGT$_v$.

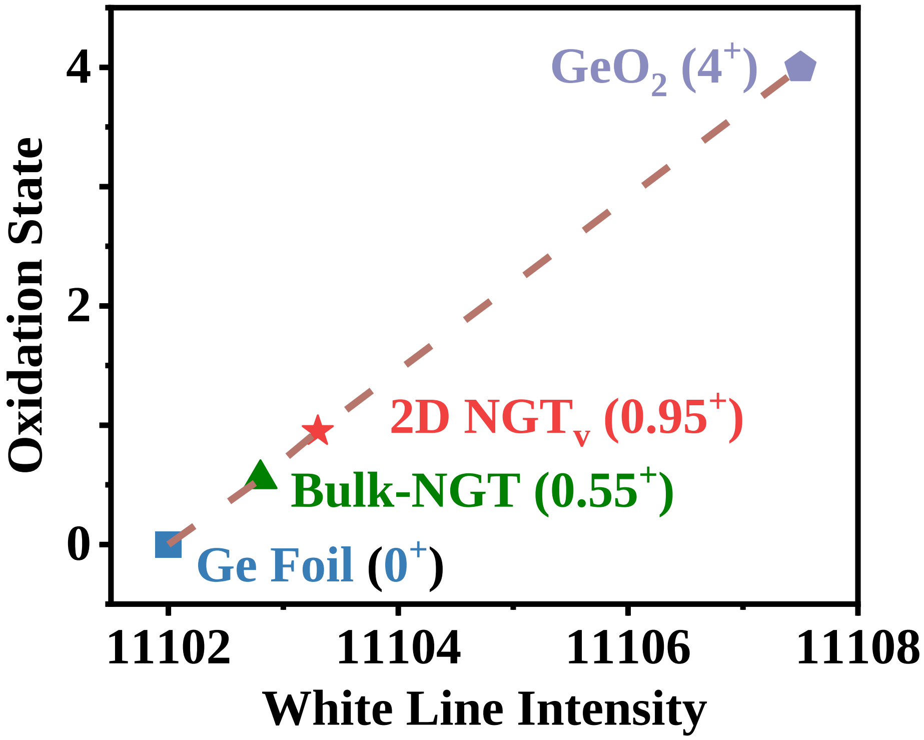

Figure S8. Average oxidation states via Ge XANES for Bulk-NGT, 2D-NGT$_v$, Ge Foil, and GeO_2.

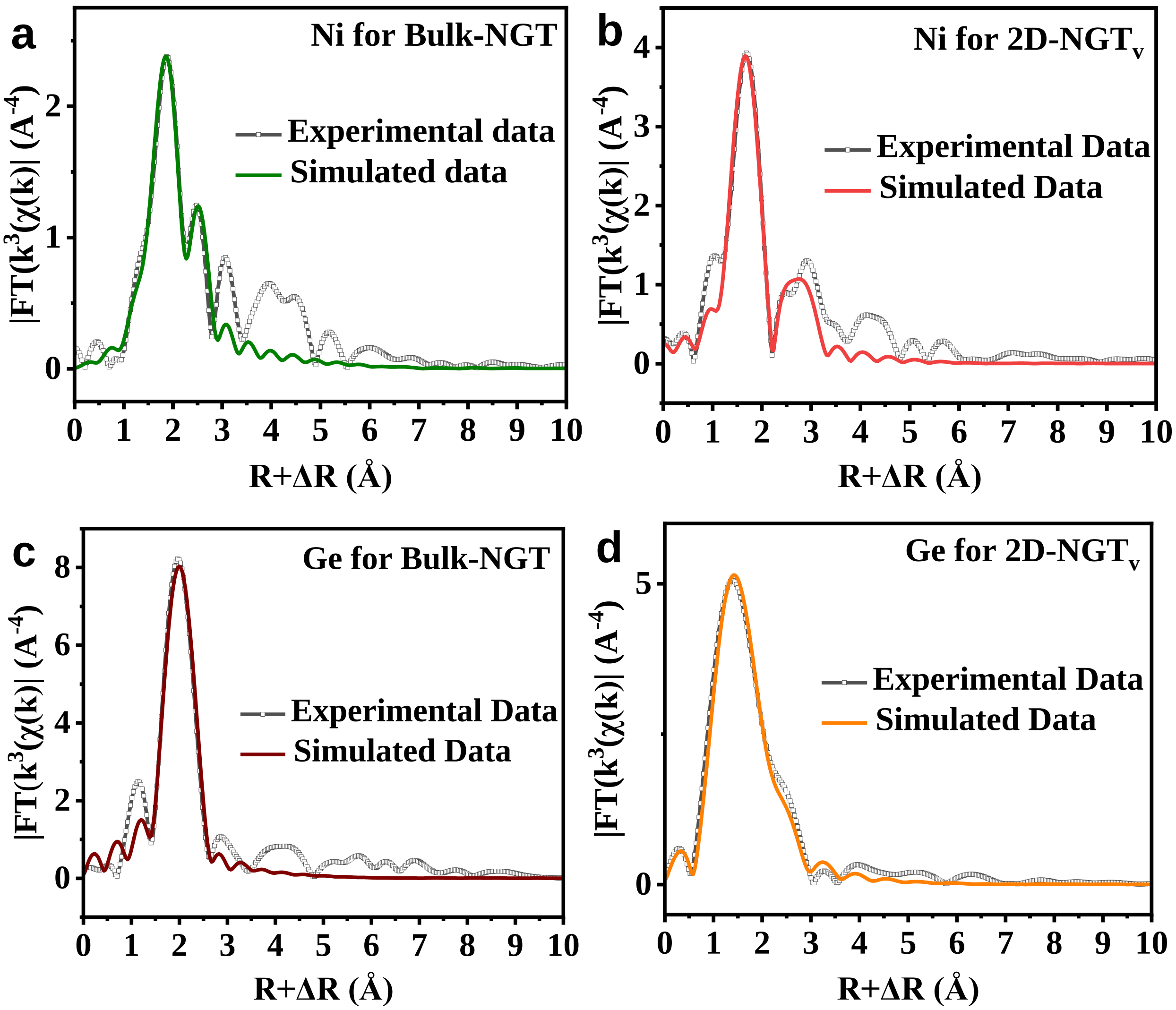

Figure S9. Ni K-edge EXAFS spectra and corresponding fitted spectra of (a) Bulk-NGT (b) 2D-NGT$_v$, Ge K-edge EXAFS spectra, and the corresponding fitted spectra (c) Bulk-NGT (d) 2D-NGT$_v$.

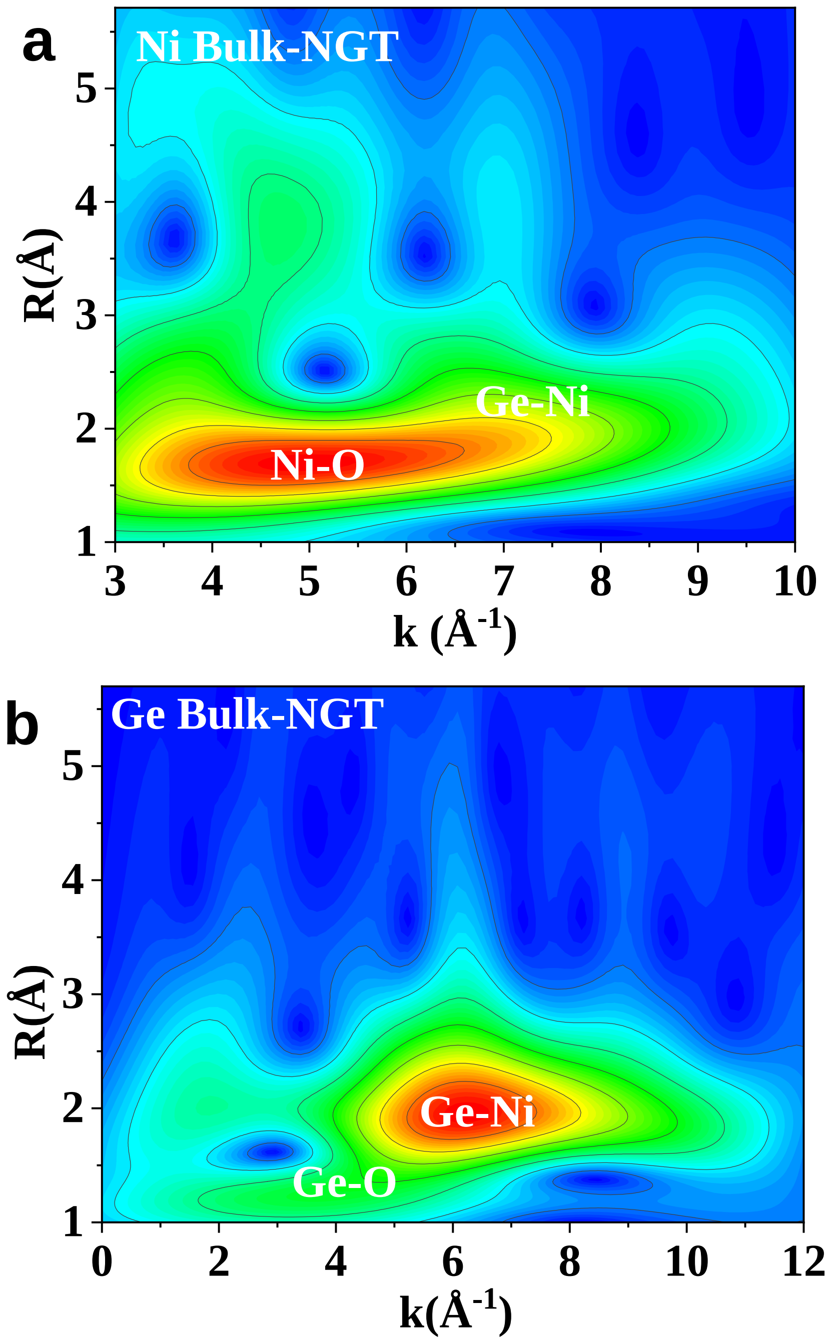

Figure S10. (a) WT for Bulk-NGT Ni K-edge. (b) WT for Bulk-NGT Ge K-edge.

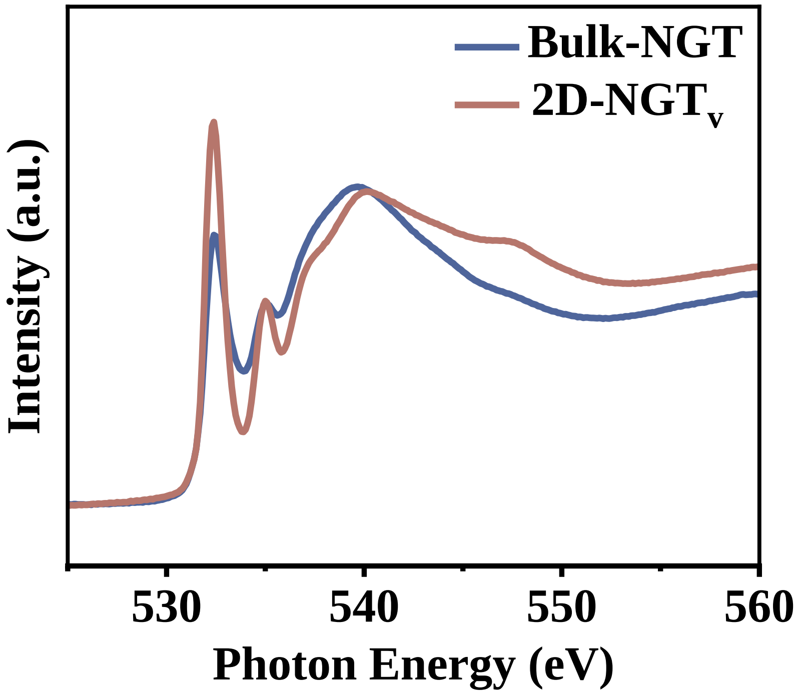

Figure S11. O K-edge profiles of Bulk-NGT and 2D-NGT$_V$.

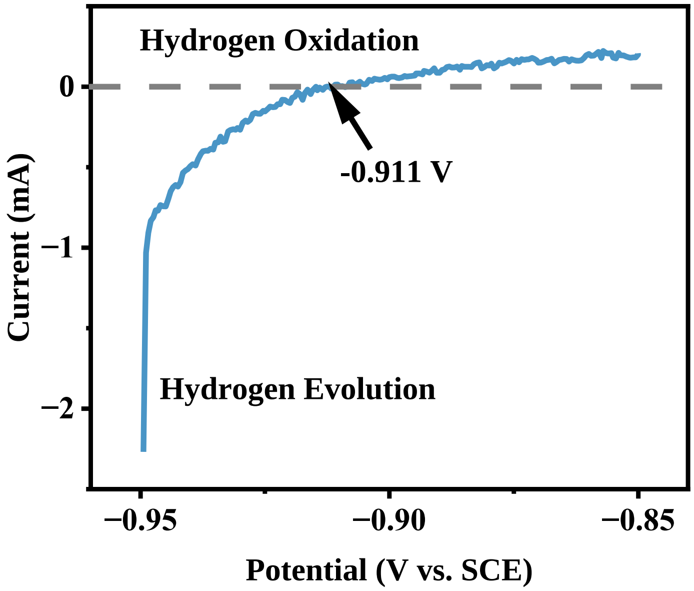

Figure S12. The LSV curve for reference electrode calibration. the calibration of the reference electrode was conducted in the 0.1 M KOH solution. Specifically, Ir blank coated onto Pt mesh was used as the working electrode and Pt wire was used as the counter electrode. The Hg/HgO electrode was employed as the reference electrode. During the calibration, pure H_2 gases (1 atm) were bubbled into the 0.1 M KOH solution until fully saturation conditions. After that, the polarization curve was captured and presented below. The standard hydrogen electrode is recognized as 0 V.

0 V. SHE = $E_{(Hg/HgO)} + E^{\circ}_{(Hg/HgO)} + (0.059 \times pH)$

$E^{\circ}_{(Hg/HgO)} = -(E_{(Hg/HgO)} + (0.059 \times pH)) = 0.11$ V *vs.* SHE

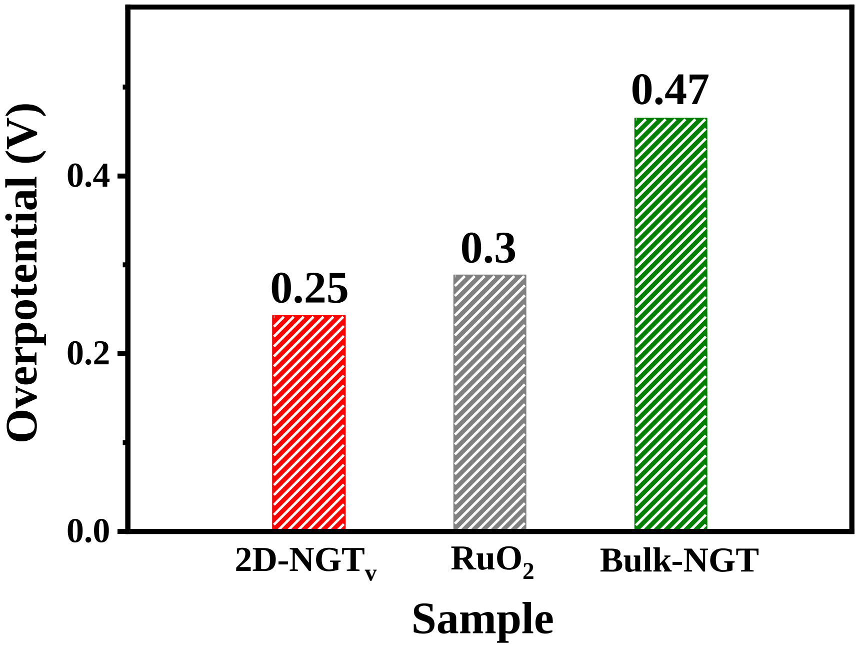

Figure S13. The Overpotential for 2D-NGT$_v$, Bulk-NGT, and RuO_2 at current density of 10 mA cm^{-2}.

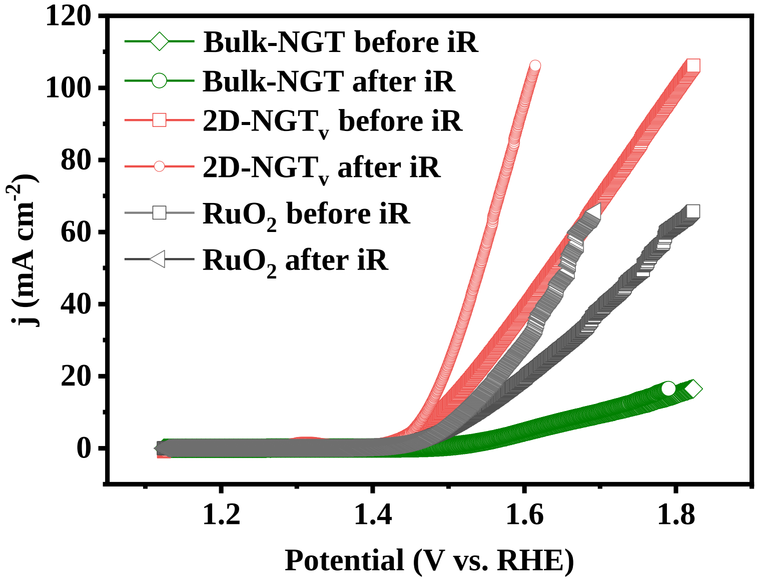

Figure S14. LSV curve of NGT catalysts and RuO_2 before and after iR feedback, the impedance of 2D-NGT$_v$, Bulk-NGT, and RuO_2 is is ~9.8 Ω, ~10.1 Ω, and ~10.5 Ω, separately.

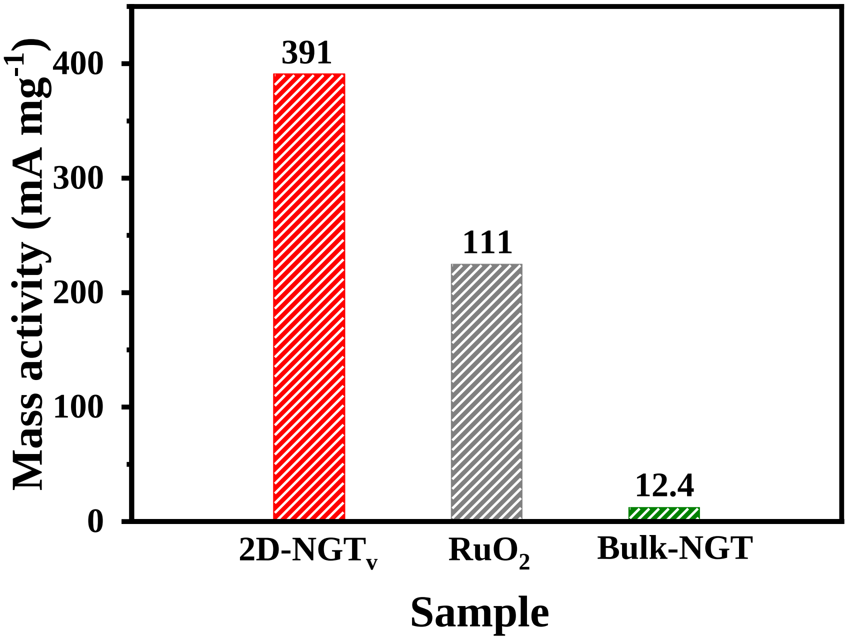

Figure S15. Mass specific activities of 2D-NGT$_v$, Bulk-NGT, and RuO_2 at overpotential of 250 mV.

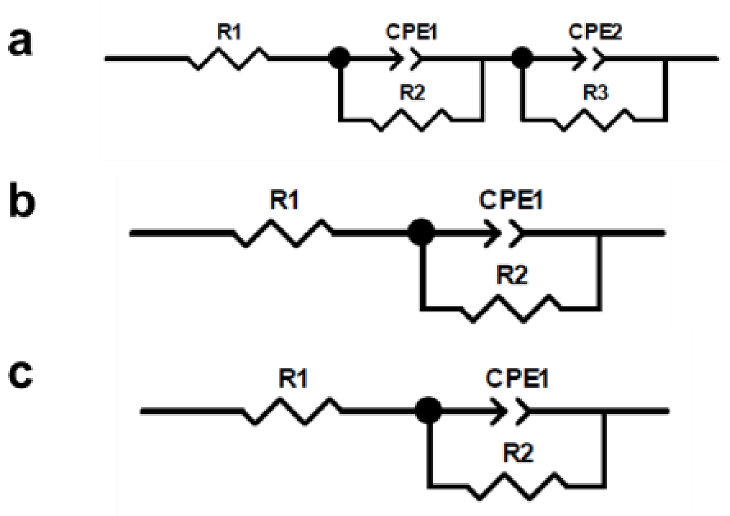

Figure S16. The equivalent circuit of 2D-NGT_v, Bulk-NGT and RuO_2. The fitted values are shown in Table S3.

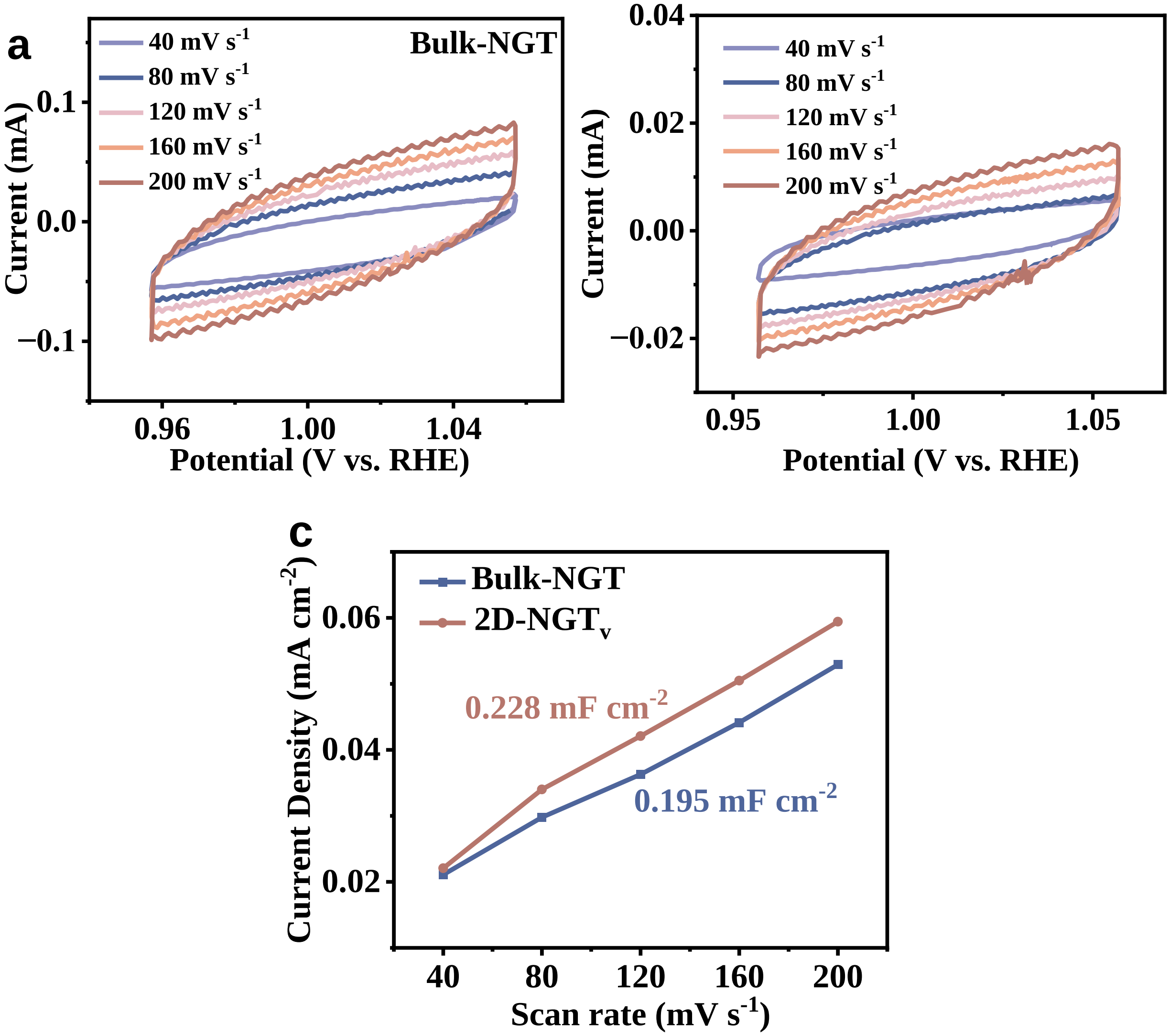

Figure S17. ECSA in 1M KOH. CV scans of (a) Bulk-NGT, (b) 2D-NGT$_v$ in 1M KOH at various scan rates between 40 mV s^{-1} and 200 mV s^{-1}. (c) Double-layer capacitance (C_{dl}) plots.

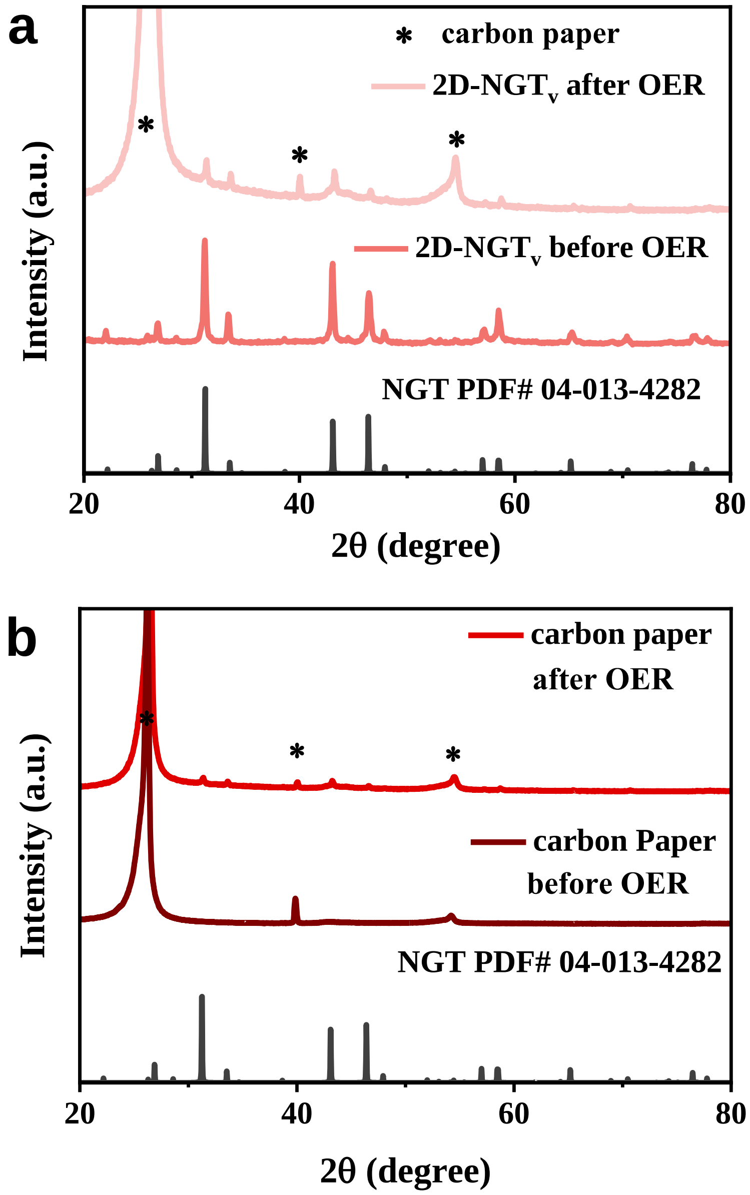

Figure S18. (a) XRD pattern 2D-NGT$_v$ after and before OER, suggesting the 2D-NGT$_v$ existing after OER. (b) The XRD pattern of 2D-NGT$_v$ after OER and Carbon paper.

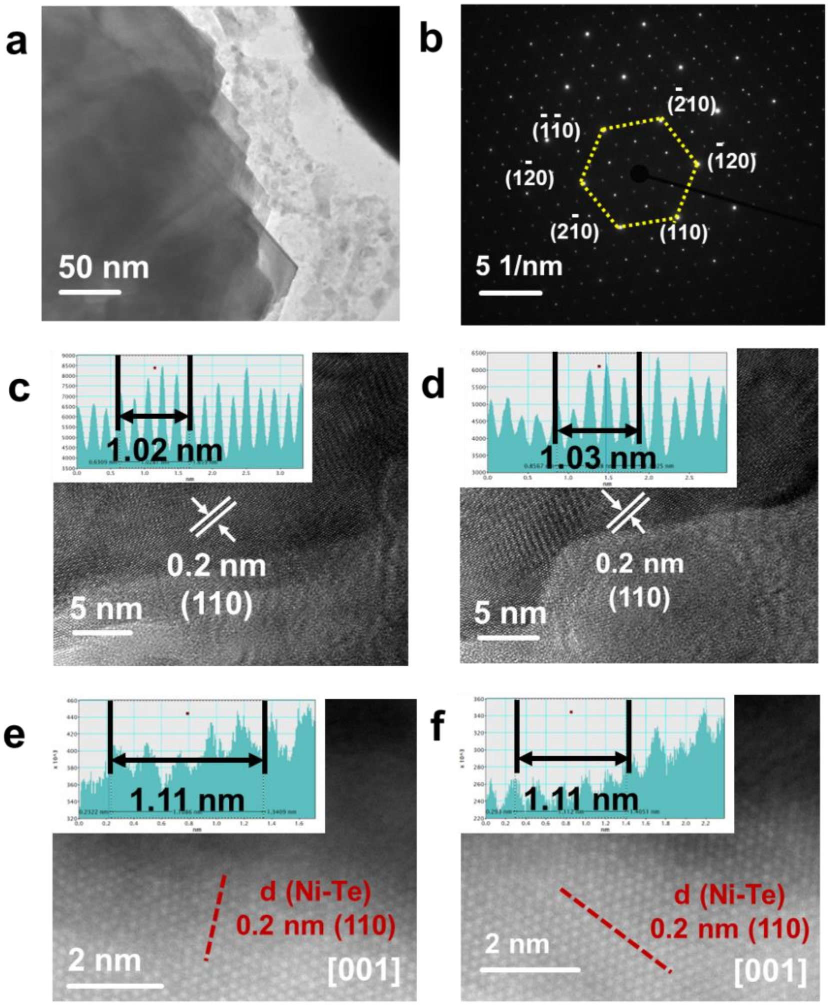

Figure S19. (a) TEM image of 2D-NGT$_v$ after OER. (b) SAED pattern of 2D-NGT$_v$ after OER. (c-d) HRTEM images of 2D-NGT$_v$ after OER. The intensity profile of the white region's line scan is presented in the inset. (e-f) HAADF-STEM images of 2D-NGT$_v$ after OER along the zone axis of [001]. The line scan intensity profile of the white area has been shown inset. It suggests the presence of Te vacancies after OER.

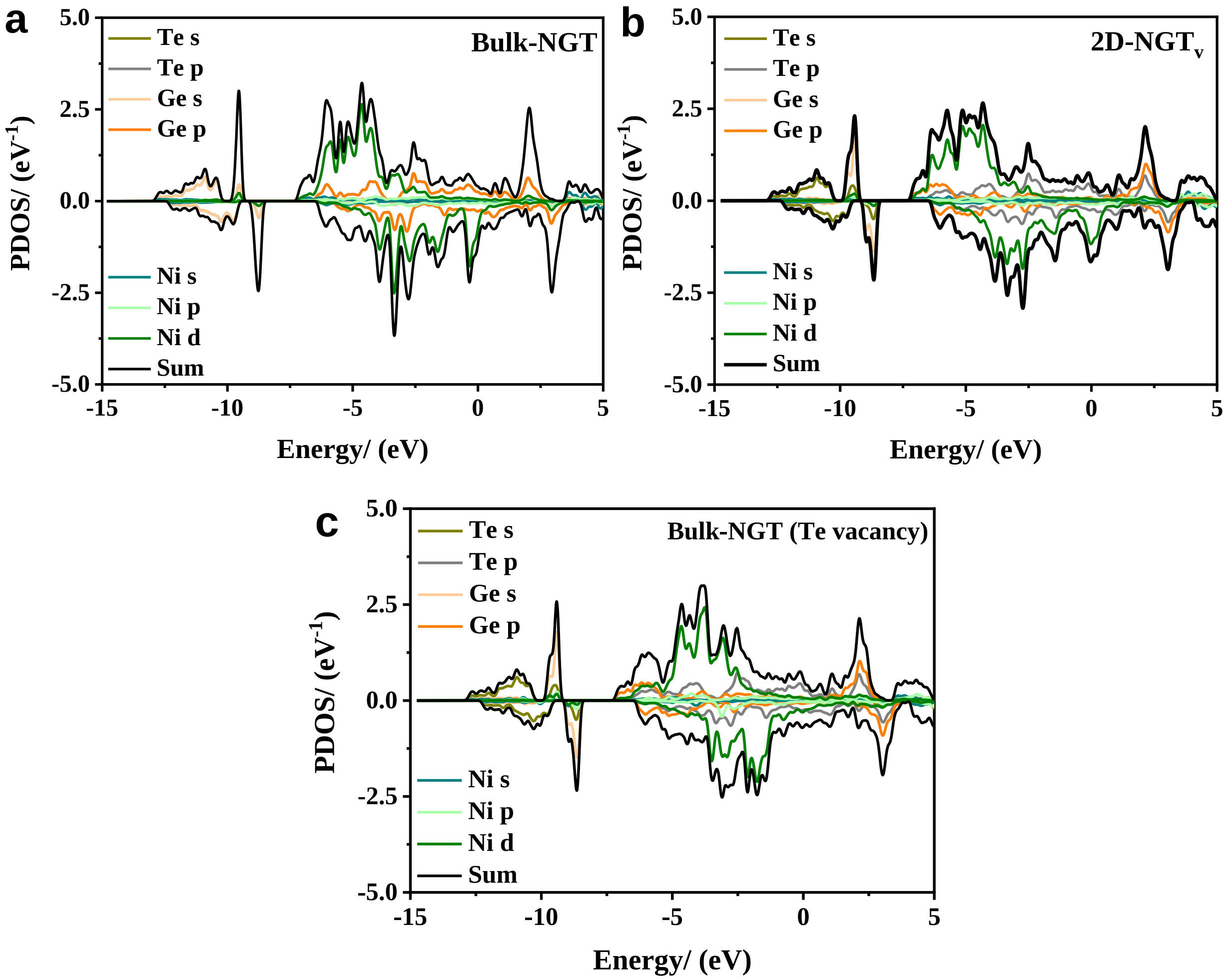

Figure S20. The calculated PDOS for (a) Bulk-NGT, (b) 2D-NGT$_v$, and (c) Bulk-NGT (Te vacancy).

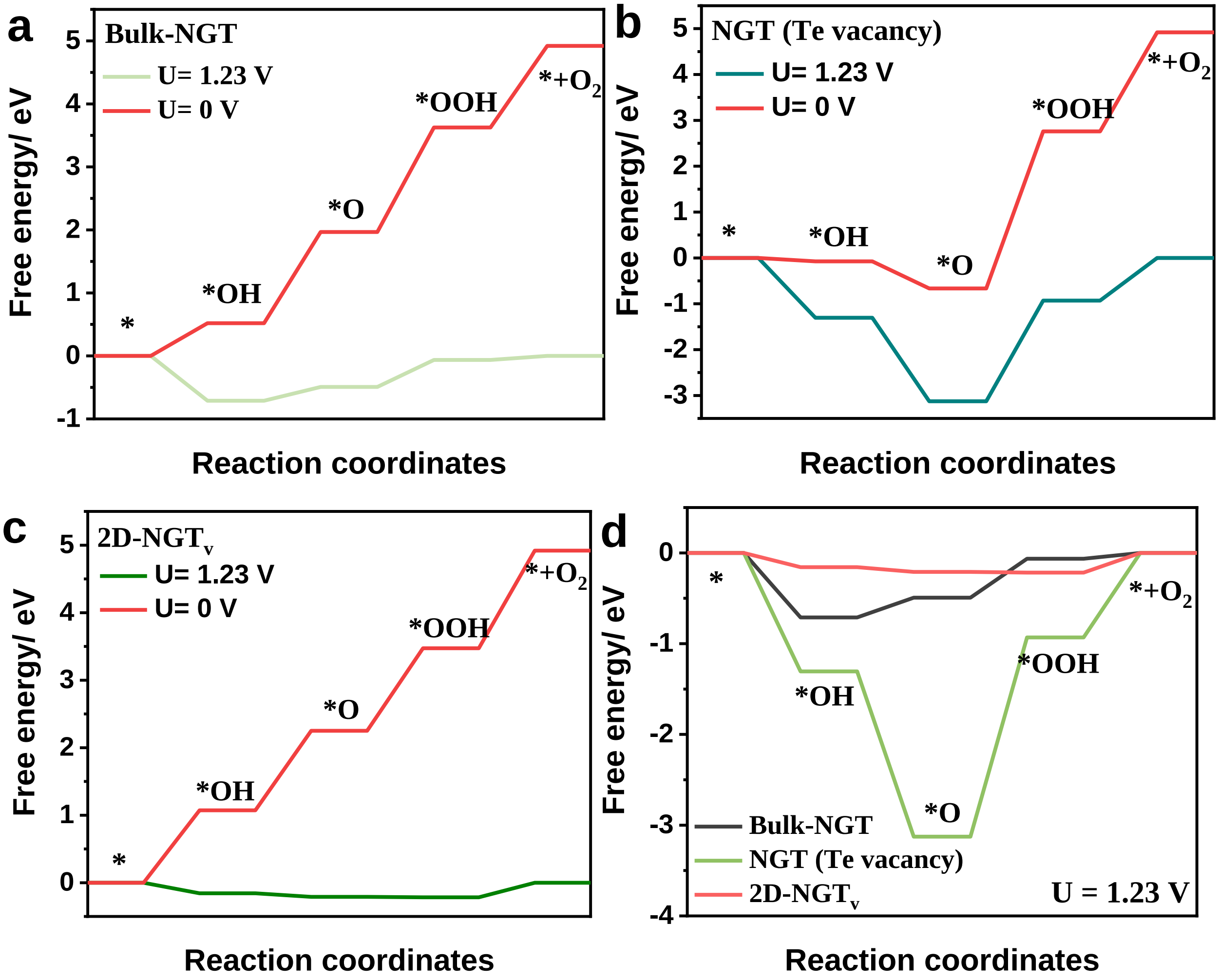

Figure S21. Free energy diagrams of OER for (a) Bulk-NGT, (b) NGT (Te vacancy), (c) 2D-NGT$_v$, (d) Free energy diagrams at the conditions of U = 0V for Bulk-NGT, NGT (Te vacancy) and 2D-NGT$_v$.

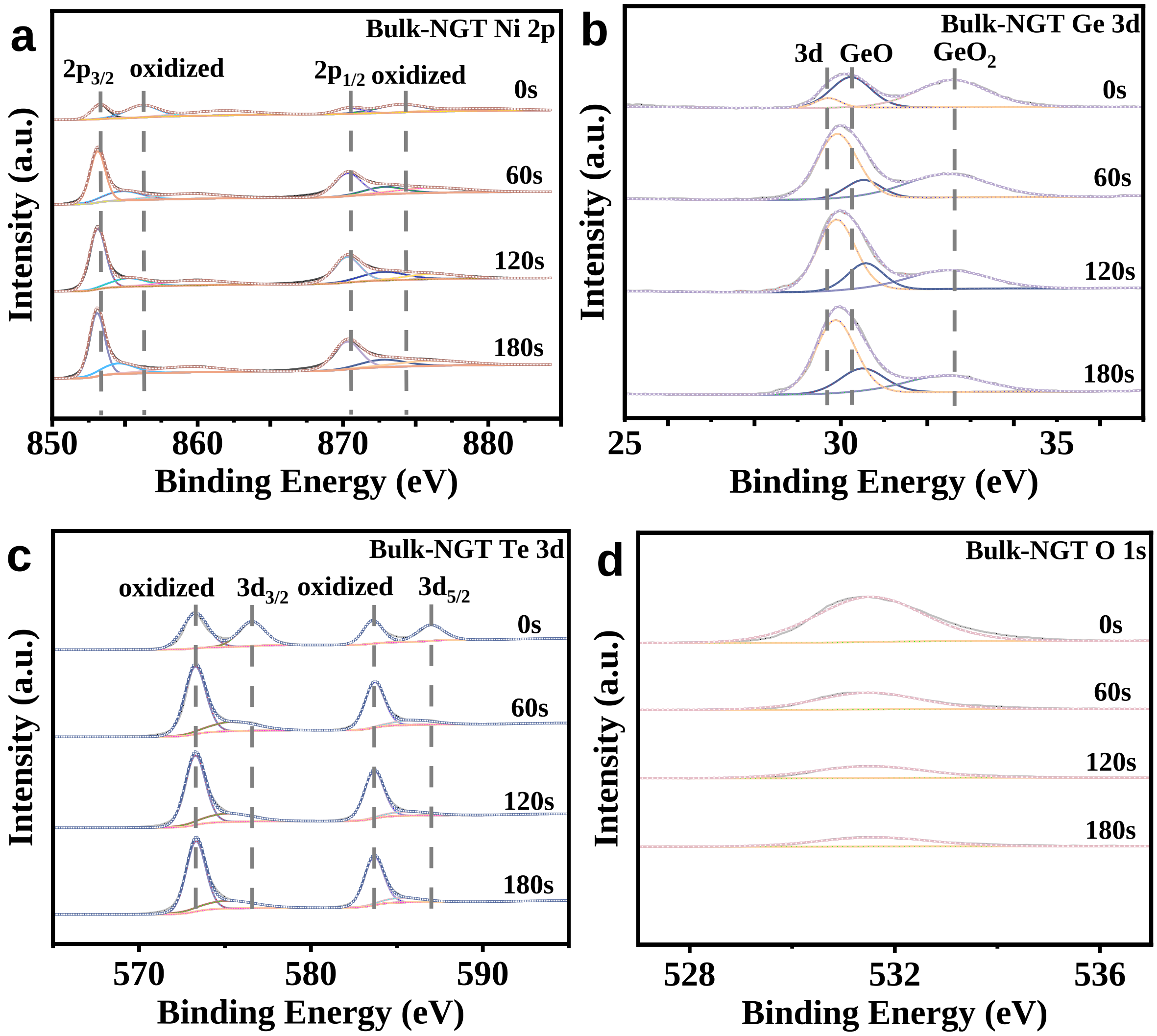

Figure S22. XPS spectra of Bulk-NGT (a) Ni 2p, (b) Ge 3d, (c) Te 3d, (d) O 1s.

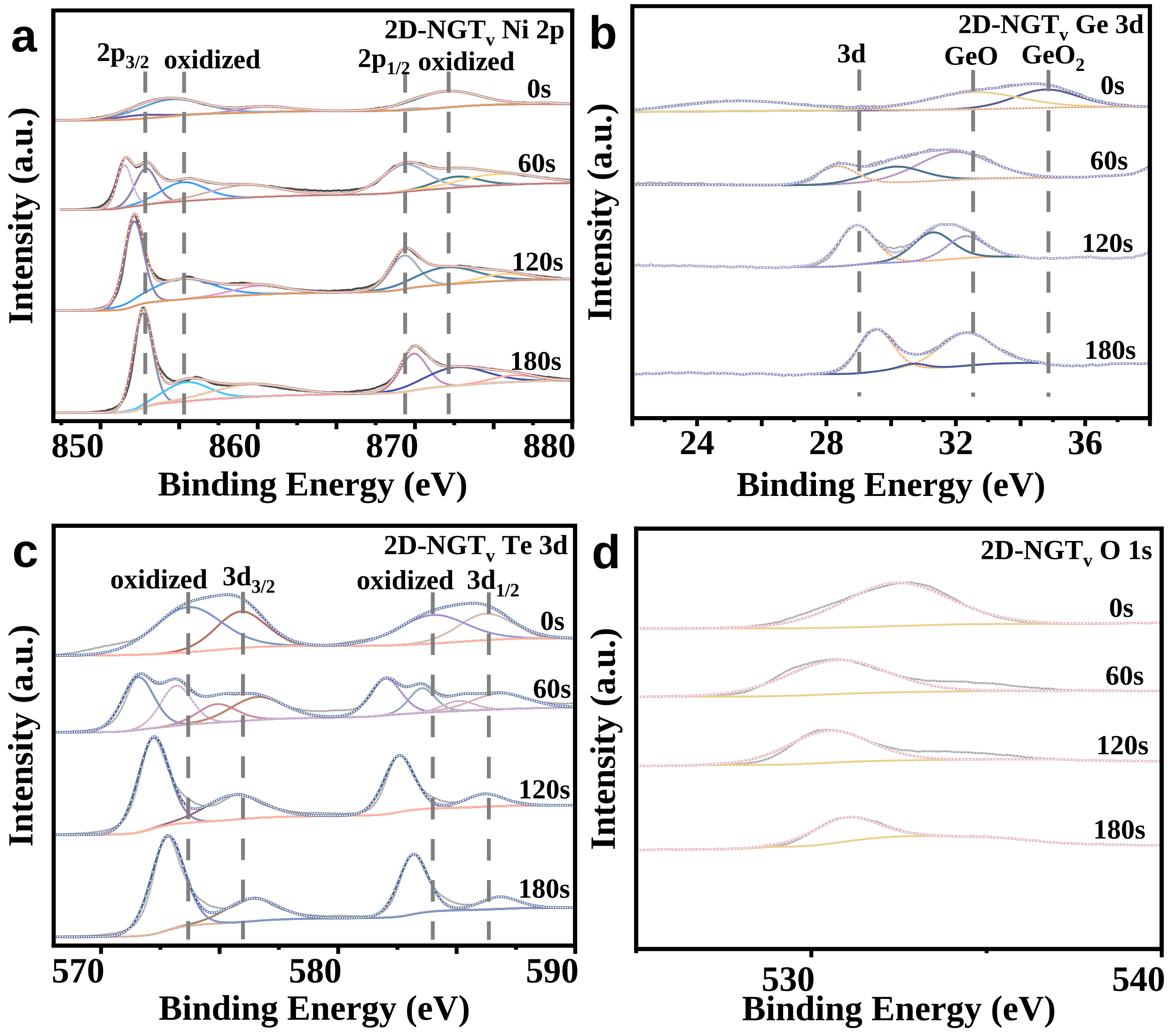

Figure S23. XPS spectra of 2D-NGT$_v$ (a) Ni 2p, (b) Ge 3d, (c) Te 3d, (d) O 1s.

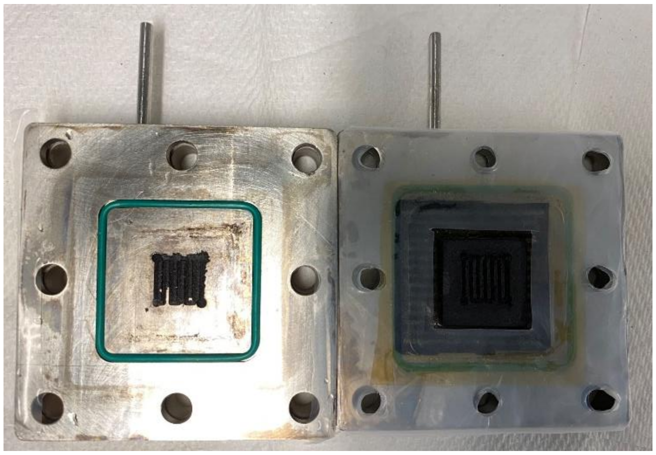

Figure S24. The optical images of the assembled anion-exchange membrane water electrolyzer components. This includes two endplates, gaskets, O-rings, and catalysts-coated membranes.

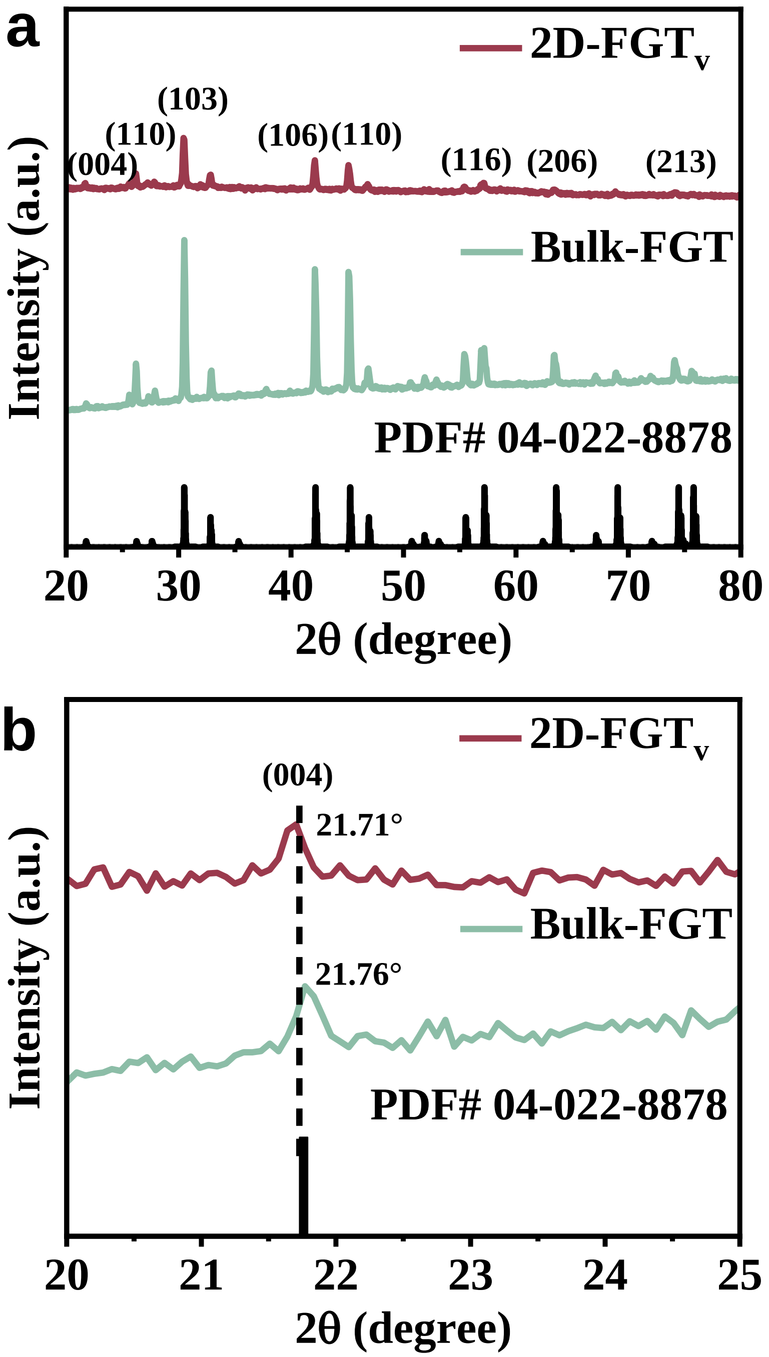

Figure S25. Bulk-FGT and 2D-FGT$_v$ (a) XRD patterns (20° - 80°). (b) The details about (004) plane of XRD pattern (20° - 25°), indicating the lattice expansion from Bulk-FGT to 2D-FGT$_v$.

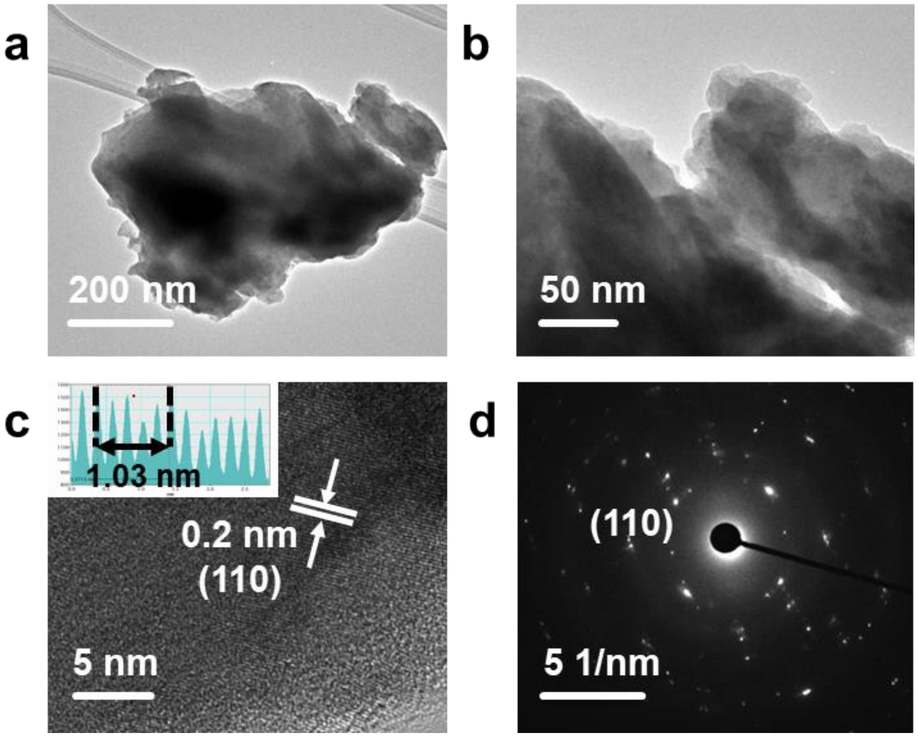

Figure S26. TEM of bulk-FGT. (a-b) Bulk-FGT TEM images. (c) HRTEM images for Bulk-FGT. (d) SAED pattern for Bulk-FGT.

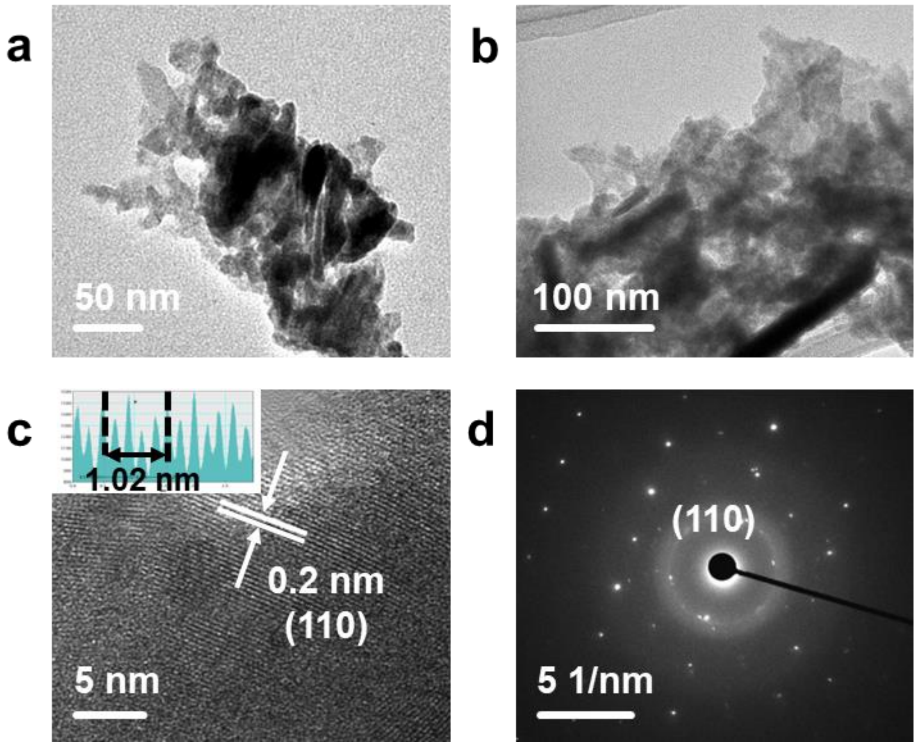

Figure S27. (a-b) TEM image of 2D-FGT$_v$, (c) HRTEM image of 2D-FGT$_v$, (d) SAED pattern of 2D-FGT$_v$.

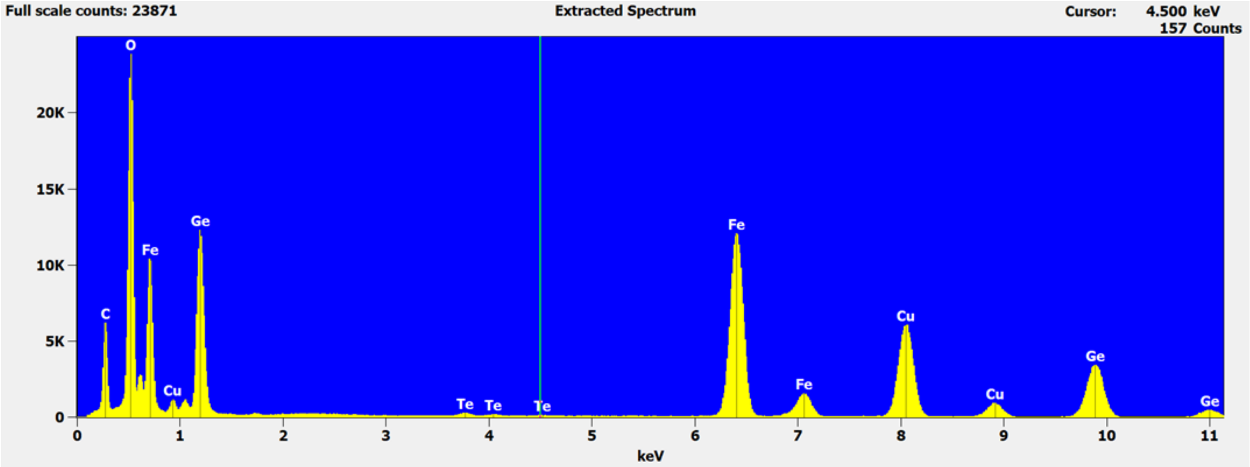

Figure S28. EDS spectrum of 2D-FGT_v indicates the existing Fe, Ge, and Te of 2D-FGT_v.

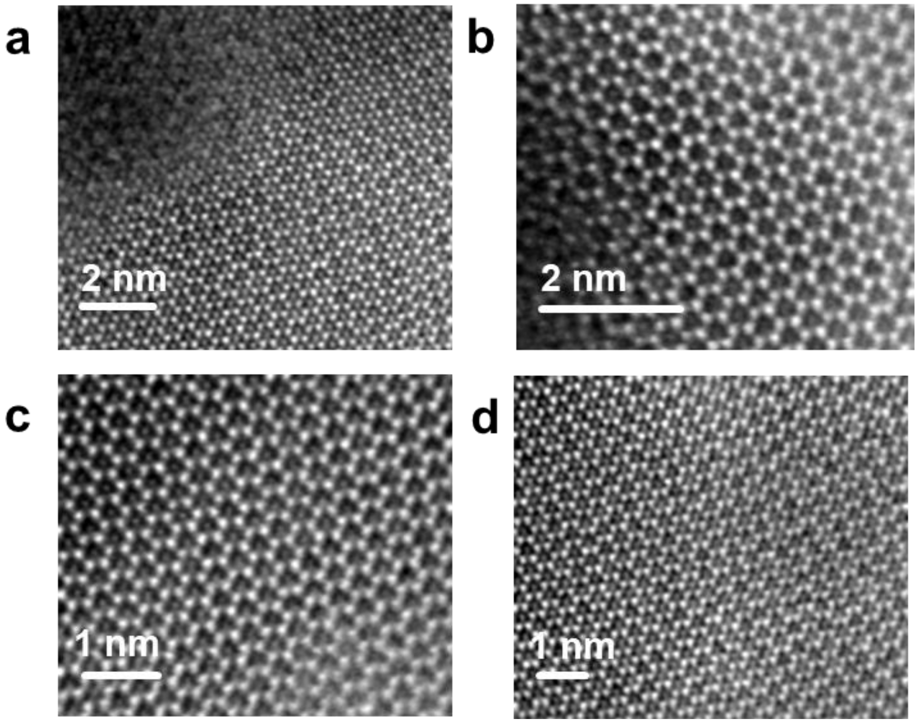

Figure S29. STEM images of 2D-FGT_v.

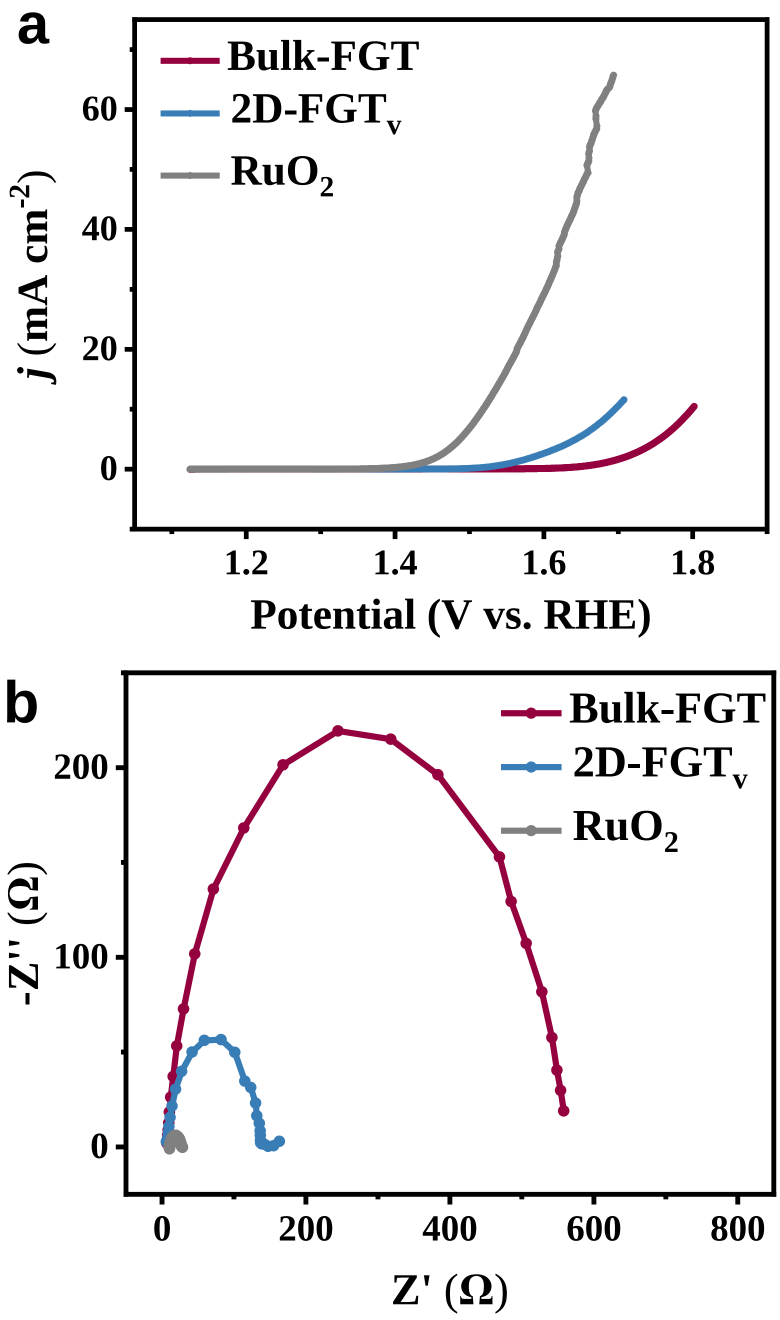

Figure S30. Alkaline OER for Bulk-FGT and 2D-FGT$_V$. (a) LSV curves for FGT catalysts and RuO_2. (b) EIS Nyquist plots of FGT and RuO_2 at 1.65 V vs RHE.

Table S1. Fitting results for Ni, Ge, Te XPS peaks for Bulk NGT and 2D-NGT_v.

XPS Peak	Bulk-NGT				2D-NGT_v			
	Peak (BE)	FWHM (eV)	Area (P) CPS.eV	Atomic (%)	Peak (BE)	FWHM (eV)	Area (P) CPS.eV	Atomic (%)
**$Ni2p_{1/2}$**	870.08	2.29	55594.2	9.83	870.38	2.6	23304.59	3.82
**$Ni2p_{1/2}$ Oxidized**	873.58	2.98	37141.17	6.6	873.98	4.21	58537.76	9.64
**$Ni2p_{1/2}$ satellite**	876.28	2.37	12157.76	2.17	879.48	4.38	17659.61	2.93
**$Ni2p_{3/2}$**	852.88	1.31	68768.43	11.92	852.78	1.62	27184.53	4.37
**$Ni2p_{3/2}$ Oxidized**	855.68	3.81	53858.77	9.37	856.08	3.87	84770.51	13.68
**$Ni2p_{3/2}$ satellite**	860.48	4.23	27805.27	4.86	861.78	4.61	41518.33	6.74
**Ge3d**	29.68	1.35	7515.59	0.7	29.48	1.17	1417.22	1.95
**GeO**	32.38	3.13	12311.77	1.14	32.48	2.52	18517.34	25.53
**GeO_2**	35.08	1.87	1905.38	0.18	34.88	1.65	1981.92	2.74
**$Te3d_{3/2}$ Oxidized**	572.88	2.02	235712.4	31.19	572.88	2.02	132233.8	7.96
**$Te3d_{3/2}$**	576.18	1.81	56646.04	7.52	576.18	3	157894	9.53
**$Te3d_{5/2}$ Oxidized**	583.28	1.38	108142.4	14.44	583.18	1.9	79978.18	4.85
**$Te3d_{5/2}$**	586.58	1.56	29591.58	0.09	586.58	3.02	102726	6.25

Table S2. EXAFS fitting results at Ni K-edge and Ge K-edge for Bulk-NGT and 2D-NGT_v.

			**Ni K-edge**			
**Sample**	shell	N	R(Å)	σ^2(Å^2)	ΔE_0(eV)	R factor
**Bulk-NGT**	Ni-Te	4.83	2.58521	0.02049	-1.705	0.0294998
**Bulk-NGT**	Ni-O	5.988	2.20137	0.04446	-1.705	0.0294998
**Bulk-NGT**	Ni-Ni	9.21	2.56593	0.02600	-1.705	0.0294998
**2D-NGT_v**	Ni-O	2.463	2.08671	0.00716	9.571	0.0111917
**2D-NGT_v**	Ni-Ni	3.488	3.09876	0.02544	9.571	0.0111917
			**Ge K-edge**			
**Sample**	shell	N	R(Å)	σ^2(Å^2)	ΔE_0(eV)	R factor
**Bulk-NGT**	Ge-Ni	4.9	2.38803	0.01142	5.22	0.0181649
**Bulk-NGT**	Ge-O	4.59	1.82658	0.05909	5.22	0.0181649
**2D-NGT_v**	Ge-O	1.696	1.76363	0.00010	3.356	0.0064027
**2D-NGT_v**	Ge-Ni	1.812	2.41574	0.00737	3.356	0.0064027

Ni K-edge: Bulk-NGT: S_0^2 was set to 0.805 for Te, 0.921 for Ni, and 0.998 for O.

2D-NGT_v: S_0^2 was set to 0.821 for O and 0.872 for Ni.

Ge K-edge: Bulk-NGT: S_0^2 was set to 0.980 for O and 0.918 for Ni.

2D-NGT_v: S_0^2 was set to 0.849 for O and 0.907 for Ni.

Table S3. The fitted values for each equivalent circuit element

Electrocatalysts	Element	Value
2D-NGT$_v$	R1	9.828
	CPE1-T	1.9889E-05
	CPE-P	0.91856
	R2	3.58
	CPE2-T	0.0035143
	CPE2-P	0.76187
	R3	3.168
Bulk-NGT	R1	10.05
	CPE1-T	5.4895E-05
	CPE-P	0.81884
	R2	98.04
RuO2	R1	10.04
	CPE1-T	0.0002643
	CPE-P	0.85384
	R2	14.96

Table S4. The Overpotential and Tafel Slope for OER electrocatalysts [1-11].

OER electrocatalysts	Overpotential (mV)	Tafel Slope (mV dec^{-1})
2D-NGT_v	245	51.19
o-$CoSe_2$-O	251	73
NiFe LDH	254	32
Co-MoS_2/BCCF	260	85
$Cu_{0.33}Co_{0.67}S_2$/CNT	284	86
$Fe_2P_2S_6$	288	45.7
CoSe@NMC	290	66.1
$CoTe_2$@N-GC	300	90
$NiPS_3$ NS	301	43
$FeCoS_2$/$Co_{0.85}$Se	330	50.8
$CoTe_2$/NF	375	68
$FePS_3$	390	58

Reference

[1] Z. Ma, C. Xiao, Z. Cui, W. Du, Q. Li, R. Sa, C. Sun, *Journal of Materials Chemistry A* **2021**, 9, 6945.

[2] Y. Zhao, J. Gu, Z. Chen, *Adv. Funct. Mater.* **2019**, 29.

[3] X. Song, Y. Qu, L. Zhao, M. Zhao, *ACS Appl. Mater. Interfaces* **2021**, *13*, 11845.

[4] Z. Wu, Y. Zhao, W. Jin, B. Jia, J. Wang, T. Ma, *Adv. Funct. Mater.* **2020**, 31.

[5] Y. Li, X. Du, J. Huang, C. Wu, Y. Sun, G. Zou, C. Yang, J. Xiong, *Small* **2019**, 15, 1901980.

[6] M. Zhao, H. J. Peng, B. Q. Li, X. Chen, J. Xie, X. Liu, Q. Zhang, J. Q. Huang, *Angew. Chem., Int. Ed.* **2020**, *59*, 9011.

[7] H.J. Deiseroth, K. Aleksandrov, C. Reiner, L. Kienle, R.K. Kremer, *Eur. J. Inorg. Chem.* **2006**, *8*, 1561.

[8] R. Gao, L. Pan, H. Wang, Y. Yao, X. Zhang, L. Wang, J.J. Zou, *Adv. Sci.* **2019**, *6*, 1900054.

[9] Z. Ma, H. Meng, M. Wang, B. Tang, J. Li, X. Wang, *ChemElectroChem,* **2018**, *5*, 335.

[10] J. G. Li, K. Xie, H. Sun, Z. Li, X. Ao, Z. Chen, K. K. Ostrikov, C. Wang, W. Zhang, *ACS Appl. Mater. Interfaces* **2019**, 11, 36649.

[11] Y. Zhao, X. Zhang, X. Jia, G.I. Waterhouse, R. Shi, X. Zhang, F. Zhan, Y. Tao, L.Z. Wu, C.H. Tung, D. O'Hare, *Adv. Energy Mater.* **2018**, *8*, 1703585.
